# Epigenetic Agents Trigger the Production of Bioactive Nucleoside Derivatives and Bisabolane Sesquiterpenes From the Marine-Derived Fungus *Aspergillus versicolor*

**DOI:** 10.3389/fmicb.2020.00085

**Published:** 2020-01-30

**Authors:** Jing-Shuai Wu, Guang-Shan Yao, Xiao-Hui Shi, Saif Ur Rehman, Ying Xu, Xiu-Mei Fu, Xiu-Li Zhang, Yang Liu, Chang-Yun Wang

**Affiliations:** ^1^Key Laboratory of Marine Drugs, The Ministry of Education of China, School of Medicine and Pharmacy, Institute of Evolution & Marine Biodiversity, Ocean University of China, Qingdao, China; ^2^Laboratory for Marine Drugs and Bioproducts, Qingdao National Laboratory for Marine Science and Technology, Qingdao, China; ^3^Institute of Oceanography, Minjiang University, Fuzhou, China; ^4^Shenzhen Key Laboratory of Marine Bioresource and Eco-Environmental Science, Shenzhen Engineering Laboratory for Marine Algal Biotechnology, College of Life Sciences and Oceanography, Shenzhen University, Shenzhen, China; ^5^Institute for Insect Biotechnology, Justus Liebig University Giessen, Giessen, Germany

**Keywords:** histone deacetylase inhibitor, DNA methyltransferase inhibitor, *Aspergillus versicolor*, nucleoside derivatives, bisabolane sesquiterpenes, antibacterial activities, antifouling activities

## Abstract

Epigenetic agents, histone deacetylase inhibitor (SAHA) and DNA methyltransferase inhibitor (5-Aza), were added to Czapek-Dox medium to trigger the chemical diversity of marine*-*derived fungus *Aspergillus versicolor* XS-20090066. By HPLC and ^1^H NMR analysis, the diversity of fungal secondary metabolites was significantly increased compared with the control. With the aid of MS/MS-based molecular networking, two new nucleoside derivatives, kipukasins K (**1**) and L (**2**) were obtained. Meanwhile, the yields of four known nucleoside derivatives were significantly enhanced. In addition, one new bisabolane sesquiterpene, aspergillusene E (**7**), along with ten known derivatives were also isolated. The structures were elucidated by comprehensive spectroscopic methods of NMR and HRESIMS analysis. Compounds **1** and **7** displayed antibacterial activities against *Staphylococcus epidermidis* and *Staphylococcus aureus* with the MIC values of 8–16 μg/mL. Our study revealed that the fungus *A. versicolor* XS-20090066 has been effectively induced by chemical epigenetic manipulation with a combination of SAHA and 5-Aza to produce new metabolites.

## Introduction

Marine-derived fungi are prolific sources of structurally novel and biologically potent natural products for drug discovery (Jin et al., 2016; Blunt et al., 2018). However, the continuous investigations on the secondary metabolites from marine-derived fungi lead to a high frequency of rediscovery of known compounds (Penesyan et al., 2010), which motivated the researchers to develop suitable strategies to find new natural products (Rutledge and Challis, 2015). A large number of fungal genome sequences suggested that the capacity of fungi to produce secondary metabolites was far more than we anticipated (Keller, 2018). Most of the biosynthetic gene clusters are silent or expressed at low level under standard laboratory conditions. Several approaches have been applied to activate the silent biogenetic gene clusters, such as OSMAC, co-culture, gene manipulation and chemical epigenetic methods (Rutledge and Challis, 2015; Keller, 2018). Recently, chemical epigenetic manipulation has been widely applied to marine-derived fungi as a handy and effective method to activate their silent gene clusters to obtain cryptic secondary metabolites (He et al., 2015; Li et al., 2017). This approach has been successful to induce or change the metabolic pathways to yield new secondary metabolites or enhance the production of the given compounds.

The fungi of *Aspergillus* genus derived from marine environment has been extensively investigated for decades due to the respected biological and pharmacological activities of their secondary metabolites (Lee et al., 2013; Wang and Ding, 2018). Chemical epigenetic manipulation has also been successfully employed to marine-derived *Aspergillus* genus to mine new cryptic secondary metabolites. For example, four new meroterpenoids were obtained from a chemical-epigenetic culture of *Aspergillus terreus* OUCMDZ-2739 with a histone deacetylase inhibitor, 10 μM trichostatin A (TSA) (Sun et al., 2018). A series of new bisabolene-type sesquiterpenoids were produced by a sea sediment-derived fungus *Aspergillus sydowii* cultured with a DNA methyltransferase inhibitor, 5-azacytidine (5-Aza) (Chung et al., 2013). Three new eremophilane-type sesquiterpenes were isolated from a marine-derived fungus *Aspergillus* sp. SCSIOW2 treated simultaneously with histone deacetylase inhibitor, suberohydroxamic acid (SBHA) and DNA methyltransferase inhibitor, 5-Aza (Wang L.Y. et al., 2016). These examples strongly proved that epigenetic manipulation is a feasible approach to induce the production of cryptic secondary metabolites from marine-derived fungi, especially *Aspergillus* genus.

In our previous studies, the fungus *Aspergillus versicolor* XS-20090066 isolated from the gorgonian *Dichotella gemmacea* collected from the Xisha Islands coral reef in the South China Sea has been proved to produce a series of bioactive anthraquinones, diketopiperazine alkaloids, phenyl ethers and nucleoside derivatives (Chen et al., 2013, 2014a,b). Furthermore, six bisabolane-type sesquiterpenoids were obtained by adding DNA methyltransferase inhibitor 5-Aza to the rice solid medium during the cultivation procedure (Wang C.Y. et al., 2016). In the present study, in order to further mine the metabolic potential of *A. versicolor* XS-20090066, this strain was co-incubated with different epigenetic agents in Czapek-Dox liquid medium. By using a combination of histone deacetylase inhibitor, suberoylanilide hydroxamic acid (SAHA) and DNA methyltransferase inhibitor, 5-Aza, the HPLC profile and ^1^H NMR spectra of the EtOAc extract of fungal culture were significantly changed comparing to the control without any epigenetic agent. A large scale fermentation with SAHA and 5-Aza led to the isolation of six nucleoside derivatives (**1**–**6**) and eleven bisabolane sesquiterpenes (**7**–**17**). Herein, we report the epigenetic manipulation of the fungal strain, and the isolation, structure elucidation and bioactivity evaluation of the isolated metabolites.

## Materials and Methods

### General Experimental Procedure

Optical rotations were recorded on a JASCO P-1020 digital polarimeter. UV spectra were determined on a HITACHI UH 5300 UV spectrophotometer. ECD data were measured by a J-815-150S Circular Dichroism spectrometer. IR spectra were acquired on a Nicolet-Nexus-470 spectrometer with a method of KBr pellets. NMR spectra of compounds **1**-**6** were recorded by a JEOL JEM-ECP NMR spectrometer (600 MHz for ^1^H NMR and 150 MHz for ^13^C NMR). While compounds **7**-**17** and EtOAc extracts of cultures were measured by a JEOL JEM-ECP NMR spectrometer (500 MHz for ^1^H NMR and 125 MHz for ^13^C NMR), HRESIMS were measured on a Thermo MAT95XP high resolution mass spectrometer, (+)-ESIMS spectra on a Thermo DSQ EImass spectrometer, and (−)-ESIMS spectra on a Bruker amaZon SL ESImass spectrometer. The HPLC analysis and purification was performed on a Hitachi L-2000 HPLC system coupled with a Hitachi L-2455 photodiode array detector and using an analytical column (Kromasil 250 mm × 4.6 mm, 5 μm) and a semi-prepared C_18_ column (Kromasil 250 mm × 10 mm, 5 μm), respectively. For HPLC analysis, the mobile phase was consisted of 0.1% formic acid in acetonitrile (A) and 0.1% formic acid in water (B) with a flowrate of 0.8 mL/min with 10 μL injection volume (2 mg/mL), and recorded at 254 nm. The elution gradient was as follows: 0 min, 5% A; 10 min, 10% A; 60 min, 100% A; 70 min, 100% A. Silica gel (Qing Dao Hai Yang Chemical Group Co.; 300-400 mesh) and Sephadex LH-20 (Amersham Biosciences) were used for column chromatography (CC). Precoated silica gel plates (Yan Tai Zi Fu Chemical Group Co.; G60, F-254) were used for thin-layer chromatography. Artificial sea salt was purchased from Zhongyan Qingdao Salt Industry Co., Ltd.

### Fungal Material

The fungus *A*. *versicolor* XS-20090066 was isolated from the fresh tissue of the inner part of the gorgonian *D. gemmacea*, which was collected from the Xisha Islands coral reef in the South China Sea in December 2009. This fungal strain was identified as *A. versicolor* according to its morphological traits and ITS sequence with the GenBank (NCBI) accession number MN880095, which had 99.82% sequence identity with 100% query cover to that of the *A. versicolor* strain ATCC 9577 (NCBI GenBank accession number AY373880). This strain was previously described as *Aspergillus* sp. XS-20090066 with NCBI GenBank accession number HM535361 (Chen et al., 2013; Wang C.Y. et al., 2016), *Aspergillus* sp. (Chen et al., 2014b), and *A. versicolor* (Chen et al., 2014a). The phylogenetic tree (Supplementary Figure S1) was constructed using the neighbor-joining method. The distance calculations, tree construction, and bootstrap analysis were performed with the software MEGA 7. This fungal strain was deposited at the Key Laboratory of Marine Drugs, the Ministry of Education of China, School of Medicine and Pharmacy, Ocean University of China, Qingdao, China.

### Molecular Networking

LC-MS/MS were analyzed on a UHPLC system (1290, Agilent Technologies) with a UPLC HSS T3 column (1.8 μm 2.1 × 100 mm, Waters) coupled to a quadrupole time-of-flight mass spectrometer 6545 (Q-TOF, Agilent Technologies) equipped with an ESI dual source in positive-ion mode. The ESI conditions were set as follows: the capillary temperature at 350°C, source voltage at 4 kV, and a sheath gas flow rate of 0.5 mL/min. The mass spectrometer was operated with an auto MS/MS mode. Mass spectra were recorded from *m/z* 50 to *m/z* 1500 in a speed of 6 spectra/sec, followed by MS/MS spectra of the twelve most intense ions from *m/z* 50 to *m/z* 1500 in a speed of 12 spectra/sec. The data were converted to mzXML format, a text-based format for mass data by using MSConvert, and uploaded to the Global Natural Products Social Molecular Networking Web site (GNPS). The parameters for generating molecular network were set with precursor mass tolerance *m*/*z* 0.02 Da, MS/MS fragment ion tolerance *m*/*z* 0.02 Da, minimum cosine score 0.7, minimum matched fragment ions 4, minimum cluster size 2, and network Top 10. The spectral library matching in GNPS was performed with the filtering parameter of identical minimum cosine score and matched fragment ion number. The generated molecular network was visualized and rearranged using Cytoscape 3.6.1.

### Fermentation, Extraction and Isolation

The fungus *A*. *versicolor* XS-20090066 was cultivated in the Czapek-Dox medium (sucrose 30 g/L, sodium nitrate 3 g/L, K_2_HPO_4_ 1 g/L, MgSO_4_⋅7H_2_O 0.5 g/L, KCl 0.5 g/L, FeSO_4_⋅7H_2_O 0.01 g/L, artificial sea salt, 30 g/L) with sixty 1 L Erlenmeyer flasks (300 mL each) by adding 100 μM SAHA or/and 100 μM 5-Aza, at room temperature for 30 days. The fermentation broth and mycelia were extracted repeatedly with equal amount of ethyl acetate (EtOAc) for three times, and concentrated *in vacuo* to give a crude extract of 53 g. The extract was subjected to silica gel CC using a step gradient elution with petroleum ether/EtOAc (10:1 to 1:4, v/v) to provide five fractions (Fr.1-Fr.5). Fr.2 was further subjected to silica gel CC with Hexane/EtOAc (10:1 to 1:2) to afford five sub-fractions (Fr.2.1-Fr.2.5). Fr.2.1 was isolated by Sephadex LH-20 CC eluted with CH_2_Cl_2_/MeOH (1:1) to provide compounds **13** (5.1 mg), **14** (4.4 mg) and **15** (4.2 mg). Fr.2.2 was purified by semi-preparative HPLC with MeOH/H_2_O (50:50) to yield **8** (2.4 mg) and **9** (3.2 mg). Fr.2.3 was subjected to semi-preparative HPLC with MeOH/H_2_O (50: 50) to yield **10** (1.7 mg). Fr.2.4 was separated by Sephadex LH-20 CC eluted with CH_2_Cl_2_/MeOH (1:1) and further purified by semi-preparative HPLC with MeOH/H_2_O (45: 55) to obtain **7** (4.2 mg), **11** (2.4 mg) and **12** (3.2 mg). Fr.3 was subjected to Sephadex LH-20 CC eluted with CH_2_Cl_2_/MeOH (1:1) to provide three sub-fractions (Fr.3.1–Fr.3.3). Fr.3.1 was isolated by semi-preparative HPLC with MeOH/H_2_O (50: 50) to obtain **16** (14.2 mg) and **17** (5.4 mg). Fr.3.2 was separated by semi-preparative HPLC with MeOH/H_2_O (30:70) to yield **5** (17 mg) and **6** (22 mg). Fr.5 was isolated on ODS CC with MeOH/H_2_O (30%→70%) to afford five fractions (Fr.5.1–Fr.5.5). Fr.5.2 was separated by Sephadex LH-20 with CH_2_Cl_2_/MeOH (1:1) and further purified by semi-preparative HPLC with MeOH/H_2_O (30:70) to produce **1** (9.3 mg) and **2** (7.1 mg). Fr.5.3 was purified by semi-preparative HPLC with MeOH/H_2_O (35:65) to give **3** (23.7 mg) and **4** (22.2 mg) (Supplementary Figure S2).

Kipukasin K (**1**): yellow oil; [α] ^20^_D_ −29.4° (*c* 1.0, MeOH); UV (MeOH) λ_max_ (log ε) 214 (3.06), 260 (2.96) nm; IR (KBr) ν_max_ 3419, 3028, 2930, 1710, 1606 1226 cm^–1^; ^1^H and ^13^C NMR data, see Table 1; HRESIMS *m/z* 543.1265 [M−H]^–^ (calcd for C_25_H_23_N_2_O_12_, 543.1256).

**TABLE 1:** ^1^H NMR and ^13^C NMR data for 1 and 2^a^.

	**1**		**2**	
**Position**	**δ_C_, type**	**δ_H_, mult (*J* in Hz)**	**δ_C_, type**	**δ_H_, mult (*J* in Hz)**
2	151.2, C		150.9, C	
4	163.5, C		163.5, C	
5	102.8, CH	5.60, d (8.1)	102.8, CH	5.57, d (8.1)
6	141.1, CH	7.67, d (8.1)	141.7, CH	7.71, d (8.1)
1′	88.9, CH	5.82, d (6.2)	87.4, CH	5.98, d (5.5)
2′	71.3, CH	4.52, d (5.7)	74.8, CH	5.39, m
3′	72.9, CH	5.32, dd (5.7, 4.0)	68.8, CH	4.36, t (5.9)
4′	79.8, CH	4.37, dt (5.5, 4.0)	82.3, CH	4.07, m
5′	64.0, CH_2_	4.44, dd (12.0, 5.5)	63.9, CH_2_	4.49, dd (12.0, 3.6)
		4.53, td (12.0, 4.0)		4.33, td (12.0, 5.9)
1″	113.9, C		113.6, C	
2″	160.4, C		160.4, C	
3″	97.5, CH	6.31, d (2.1)	97.5, CH	6.25, d (1.8)
4″	159.0, C		159.1, C	
5″	109.6, CH	6.26, d (2.1)	109.6, CH	6.20, d (1.8)
6″	138.7, C		138.7, C	
7″	167.1, C		166.8, C	
8″	20.1, CH_3_	2.21, s	20.1, CH_3_	2.14, s
9″	56.2, CH_3_	3.70, s	56.2, CH_3_	3.62, s
1″′	120.4, C		120.4, C	
2″′	116.9, CH	7.40, d (2.1)	116.9, CH	7.33, d (2.1)
3″′	145.9, C		145.8, C	
4″′	151.6, C		151.5, C	
5″′	116.0, CH	6.83, d (8.3)	115.9, CH	6.78, d (8.3)
6″′	122.5, CH	7.35, dd (8.3, 2.1)	122.5, CH	7.31, dd (8.3, 2.1)
7″′	166.0, C		166.0, C	

*^*a*^600 MHz for ^1^H NMR and 150 MHz for ^13^C NMR in DMSO-*d*_*6*_.*

Kipukasin L (**2**): yellow oil; [α] ^20^_D_ −26.5° (*c* 1.0, MeOH); UV (MeOH) λ_max_ (log ε) 216 (3.24), 257 (3.16) nm; IR (KBr) ν_max_ 3420, 3028, 2927, 1712, 1610, 1234 cm^–1^; ^1^H and ^13^C NMR data, see Table 1; HRESIMS *m/z* 543.1265 [M−H]^–^ (calcd for C_25_H_23_N_2_O_12_, 543.1256).

Aspergillusene E (**7**): white powder; UV (MeOH) λ_max_ (log ε) 219 (1.96), 250 (1.36), 266 (1.52), 294 (1.04) nm; IR (KBr) ν*_max_* 3315, 1623, 1453, 1258 cm^–1^; ^1^H and ^13^C NMR data, see Table 2; HRESIMS *m/z* 215.1428 [M−H_2_O + H]^+^ (calcd for C_15_H_19_O, 215.1430).

**TABLE 2:** ^1^H NMR and ^13^C NMR data for 7^a^.

**Position**	**δ_C_, type**	**δ_H_, mult (*J* in Hz)**
2	154.5, C	
3	109.5, C	
3a	129.1, C	
4	118.7, CH	7.40, d (7.8)
5	121.4, CH	7.15, dd (7.8, 1.5)
6	138.8, C	
7	108.9, CH	7.34, d (1.5)
7a	153.8, C	
8	24.0, CH_2_	2.73, t (7.1)
9	37.2, CH_2_	1.53, m
10	27.5, CH	1.53, m
11	22.7, CH_3_	0.91, d (5.4)
12	22.7, CH_3_	0.91, d (5.4)
13	8.1, CH_3_	2.13, s
14	63.5, CH_2_	4.56, s

*^*a*^500 MHz for ^1^H NMR and 125 MHz for ^13^C NMR in DMSO-*d*_*6*_.*

### Biological Assay

Antibacterial activities were evaluated against six pathogenic bacterial strains, *Staphylococcus epidermidis* ATCC 12228, *Staphylococcus aureus* ATCC 25923, *Pseudomonas aeruginosa* ATCC 27853, *Bacillus cereus* ATCC 14579, *Escherichia coli* ATCC 25922, and *Sarcina lutea* ATCC 9341, using a broth micro dilution method according to the standards and guidelines recommended by Clinical and Laboratory Standards Institute (CLSI, 2012). Vancomycin was used as a positive control.

Antifungal activities were tested against *Candida albicans* ATCC 24433, *Candida tropicalis* ATCC 20962 and *Candida parapsilosis* ATCC 22019 using a broth micro dilution method according to the standards and guidelines recommended by CLSI (CLSI, 2012). Amphotericin B was used as a positive control.

The cytotoxic activities were evaluated by the SRB method (Skehan et al., 1990) using five human tumor cell lines A549, HCT116, MCF-7, Hela, and Hep G2. Adriamycin was used as a positive control.

The antifouling assay was performed using bryozoan larvae of *Bugula neritina* according to the method described by Xu et al. (2010). All of the isolated compounds were screened at a concentration of 25 μg/mL to test their preliminary inhibitory effects on larval settlement. Then the active compounds were further examined to measure their EC_50_ and LC_50_ values with a 2-fold dilution method. 5-Octylfuran–2(5H)-one (butenolide) and seanine 211 was used as a positive control.

## Results

### Chemical Epigenetic Manipulation of *A. versicolor* XS-20090066

**FIGURE 1:**
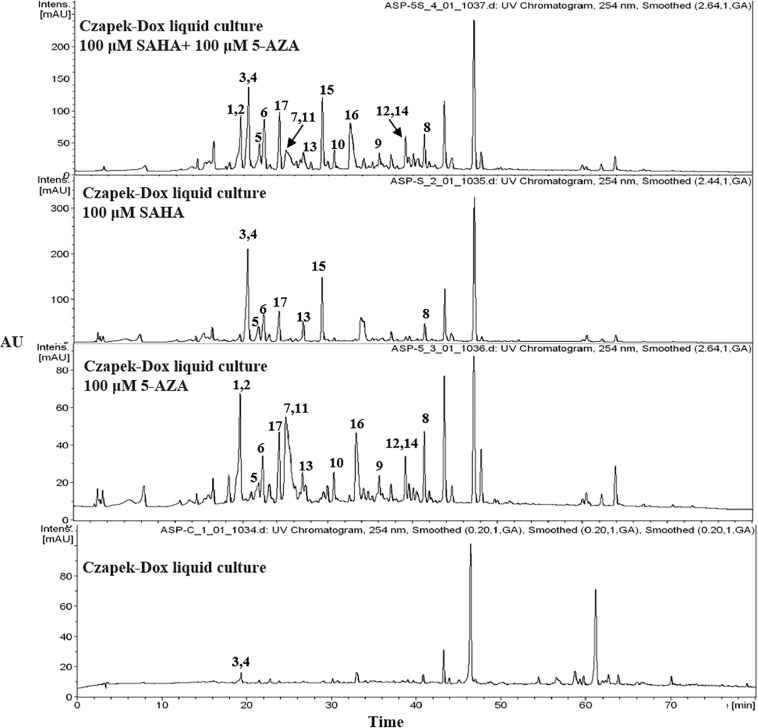
HPLC profiles of EtOAc extracts of *A*. *versicolor* XS-20090066 with epigenetic agents.

**FIGURE 2:**
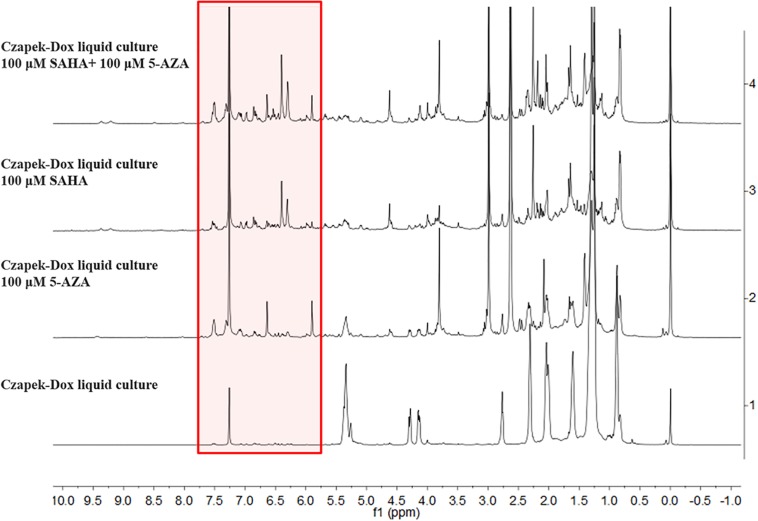
^1^H NMR spectra of EtOAc extracts of *A*. *versicolor* XS-20090066 measured in CDCl_3_ with 500 MHz, chemical shifts (δ) presented in ppm.

**FIGURE 3:**
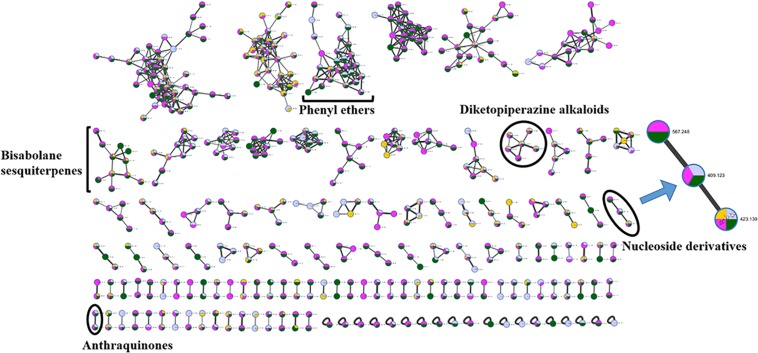
Molecular networking of *A*. *versicolor* XS-20090066 treated with SAHA (yellow), 5-Aza (pink) and both of them (green), as well as the control (gray). The clusters marked are annotated as the GNPS (Global Natural Products Social Molecular Networking Web site) library matched compounds and their derivatives, and the cluster of interest is enlarged. The thickness of edges between the nodes indicates the degree of similarity between their respective MS^2^ spectra.

**FIGURE 4:**
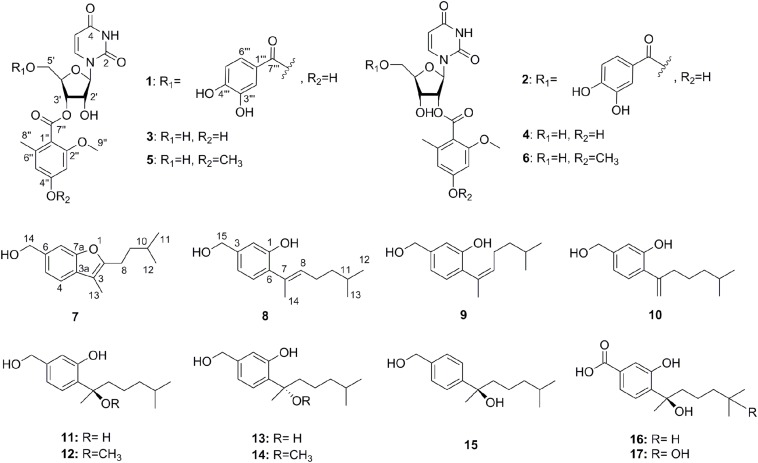
The structures of compounds **1**–**17.**

The chemical epigenetic manipulation on *A*. *versicolor* XS-20090066 was conducted in Czapek-Dox liquid medium by using histone deacetylase inhibitors (SAHA, SBHA, nicotinamide, and sodium butyrate) or/and DNA methyltransferase inhibitors (5-Aza and 2′-deoxy-5-Aza) in different concentrations (1–1000 μM), while cultivation without epigenetic modifiers was used as the control. By comparing of HPLC and ^1^H NMR analysis, cultivation with 100 μM SAHA and 100 μM 5-Aza, respectively, displayed remarkable chemical diversity of the secondary metabolites (Figures 1, 2). Furthermore, the concomitant of two inhibitors with the optimal combination of 100 μM SAHA and 100 μM 5-Aza led to an additive effect on the epigenetic regulation of the fungal metabolites as shown in the HPLC profile at *R*_t_ 18–30 min (Figure 1). In the ^1^H NMR profiles of the cultures, multiple resonations of hydrogen signals from 6.0 to 8.0 ppm belonging to the characteristic signals of unsaturated aromatic hydrogens were observed from the EtOAc extracts of the epigenetic manipulated cultures compared with the control (Figure 2). Intriguingly, the culture with a combination of SAHA and 5-Aza achieved the strongest cumulative signals compared to the cultures treated with SAHA or 5-Aza separately. The molecular networking profiles revealed that diverse structures existed in the culture, including nucleoside derivatives, anthraquinones, diketopiperazine alkaloids, phenyl ethers, bisabolane sesquiterpenes, and other unidentified secondary metabolites (Figure 3). Compared to the control, new nodes induced by epigenetic manipulation were observed, especially treated with SAHA and 5-Aza simultaneously (Figure 3). Consequently, a large scale fermentation with Czapek-Dox liquid medium was carried out with adding both 100 μM SAHA and 100 μM 5-Aza. From the EtOAc extract, 17 compounds were isolated (Figure 4), including two new nucleoside derivatives, kipukasins K (**1**) and L (**2**), and one new bisabolane sesquiterpene, aspergillusene E (**7**), along with four known nucleoside derivatives, kipukasin I (**3**) (Chen et al., 2014a), kipukasin H (**4**) (Jiao et al., 2007), kipukasins D (**5**) (Jiao et al., 2007), and kipukasins E (**6**) (Chen et al., 2014a), and ten known bisabolane sesquiterpenes, (*E*)-5-(hydroxymethyl)-2-(6′-methylhept-2′-en-2′-yl)phenol (**8**) (Sumarah et al., 2011), (*Z*)-5-(hydroxymethyl)-2-(6′-methylhept-2′-en-2′-yl)phenol (**9**) (Sumarah et al., 2011), 7-deoxy-7,14-didehydrosydonol (**10**) (Chung et al., 2013), (7*R*)-(−)-sydonol (**11**) (Li et al., 2012), (7*R*)-(−)-methoxyethanol (**12**) (McMullin et al., 2015), (7*S*)-(+)-sydonol (**13**) (Nukina et al., 1981), (7*S*)-(+)-methoxyethanol (**14**) (Chung et al., 2013), aspergiterpenoid A (**15**) (Li et al., 2012), sydonic acid (**16**) (Hamasaki et al., 1978), and hydroxysydonic acid (**17**) (Hamasaki et al., 1978).

### Structure Elucidation

**FIGURE 5:**
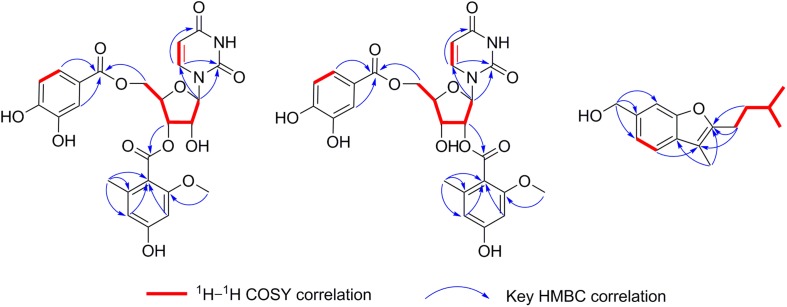
The key ^1^H–^1^H COSY and HMBC correlations of compounds **1, 2,** and **7**.

Kipukasin K (**1**) was isolated as a yellow oil, and the molecular formula was determined as C_25_H_24_N_2_O_12_ based on its (−)-HRESIMS at *m/z* 543.1265 [M - H]^–^ (calcd 543.1256), indicating 15 degrees of unsaturation. The planar structure was elucidated by the analysis of 1D/2D NMR (Supplementary Figures S3–S11). One 1,2-disubstituted olefin group (δ_H_ 5.60 (d, *J* = 8.1 Hz), δ_C_ 102.8 and δ_H_ 7.67 (d, *J* = 8.1 Hz), δ_C_ 141.1), and two amide carbonyls (δ_C_ 151.2 and δ_C_ 163.5) were observed in the ^1^H and ^13^C NMR spectra. One pentose moiety (δ_H_ 4.37 to 5.82, δ_C_ 64.0 to 88.9) was elucidated on the basis of the *J*-values (Table 1) and ^1^H–^1^H COSY of H-1′ to H-5′ (Figure 5). These NMR spectroscopic characteristics were similar to those of the typical uridine analog. The HMBC correlations from H-1′ to C-2/C-6 indicated that the pentose moiety was connected to N-1 (Figure 5). Careful analysis of the ^1^H and ^13^C NMR revealed the presence of 1,2,4,6-tetra-substituted [δ_H_ 6.26 (d, *J* = 2.1 Hz) and δ_H_ 6.31 (d, *J* = 2.1 Hz)] and 1,3,4-trisubstituted benzene [δ_H_ 6.83 (d, *J* = 8.3 Hz), δ_H_ 7.35 (dd, *J* = 8.3, 2.1 Hz), and δ_H_ 7.40 (d, *J* = 2.1 Hz)] moieties, as well as two ester carbonyls (δ_C_ 166.0 and δ_C_ 167.1). One methyl and one methoxyl were anchored at C-6″ and C-2″ of the 1,2,4,6-tetra-substituted benzene, respectively, based on the HMBC correlations from H-8″ to C-1″/C-5″/C-6″ and from H-9″ to C-2″. The above spectroscopic features of **1** were very similar to those of the co-isolated known nucleoside derivative kipukasin I (**3**). The 1,2,4,6-tetra-substituted aroyl unit was connected to C-3′ of the sugar moiety based on the HMBC correlation from H-3′ to C-7″. The obvious difference was an additional 1,3,4-trisubstituted aroyl unit (protocatechuic ester moiety) in **1**. The HMBC correlation from H-5′ to C-7″′ suggested this moiety was located at C-5′.

Kipukasin L (**2**) was also isolated as a yellow oil. The molecular formula was established as C_25_H_24_N_2_O_12_ with 15 degrees of unsaturation based on the (−)-HRESIMS at *m/z* 543.1265 [M - H]^–^ (calcd 543.1256). The ^1^H and ^13^C NMR spectra of **2** were very similar to those of **1** (Supplementary Figures S12–S20). The main difference was the chemical shifts of H-2′ (δ_H_ 5.39 in **2** vs. δ_H_ 4.52 in **1**) and H-3′ (δ_H_ 4.36 in **2** vs. δ_H_ 5.32 in **1**); C-2′ (δ_C_ 74.8 in **2** vs. δ_C_ 71.3 in **1**) and C-3′ (δ_C_ 68.8 in **2** vs. δ_C_ 72.9 in **1**). It was suggested that the difference was the location of the tetra-substituted aroyl group. The HMBC correlation from H-2′ to C-7″ in **2** supported that it was located at C-2′ rather than C-3′ of ribose unit in **2** (Figure 5). Therefore, **2** is the regioisomer of **1**.

The ROESY correlations between H-6 and H-1′/H-2′, and H-1′ and H-4′ in compounds **1** and **2** were observed, suggesting that H-6, H-1′, H-2′ and H-4′ should be at the same side (Supplementary Figure S21). However, no correlation between 1,3,4-trisubstituted aroyl protons and 1,2,4,6-tetra-substituted aroyl protons or between aroyl protons and uracil olefin protons was observed from their ROESY spectra (Supplementary Figures S8, S17). Comparison of the chemical shifts of H-1′ (δ_H_ 5.82 in **1** and δ_H_ 5.98 in **2**) and their coupling constants (6.2 Hz in **1** and 5.5 Hz in **2**) with the reported ribose units confirmed that the sugar moieties in **1** and **2** were β-ribose (Ohigashi et al., 1989; Ostrowski et al., 2003). The absolute configurations of **1** and **2** were speculated based on the biogenetic considerations. The uridine unit could be established as uracil-1-β-D-ribofuranoside, identical to those of the co-isolated known nucleoside derivatives **3**–**6** (Jiao et al., 2007).

Aspergillusene E (**7**) was obtained as a white powder. The molecular formula was determined as C_15_H_20_O_2_ with six degrees of unsaturation on the basis of its (+)-HRESIMS at *m/z* 215.1428 [M - H_2_O + H]^+^ (calcd 215.1430). The UV spectrum showed absorption bands of a benzofuran chromophore at 219, 250, 266, and 293 nm (Trofast, 1978). The ^1^H NMR spectrum revealed the presence of one 1,2,4-trisubstituted benzene, one hydroxyl, three methylenes (one oxygenated), one methine, and three methyl groups (Table 2 and Supplementary Figure S22). The ^13^C NMR spectrum displayed the presence of 15 carbons, containing eight olefinic carbons (two oxygenated), one methine carbon, three methylene carbons (one oxygenated) and three methyl carbons (Supplementary Figures S23, S24). The isopentyl side chain, established by the ^1^H–^1^H COSY of H-8/H-9/H-10/H-11/H-12, was connected to C-2 based on the HMBC correlations from H-8 to C-2/C-3 and from H-9 to C-2 (Figure 5 and Supplementary Figures S25, S26). As the presence of one benzene group and one olefinic group in the molecule occupied five degrees of unsaturation, another ring should exist in **7**, indicating a benzofuran moiety formed between C-2 and C-7a through one oxygen atom. The HMBC correlations from H-13 to C-2/C-3a revealed that the methyl was located at C-3. These spectroscopic characteristics were similar to the reported aspergillusene B, a bisabolane sesquiterpene from *A. sydowii* PSU-F154 (Trisuwan et al., 2011), except the oxygenated methylene group in **7** instead of a carboxyl group (Supplementary Figures S27, S28). The HMBC correlations from H-14 to C-5, C-6, and C-7 suggested that the oxygenated methylene was anchored at C-6 (Figure 5).

In our previous studies on this strain, four known nucleoside derivatives (**3**-**6**) were isolated from its rice culture (Chen et al., 2014a). By adding 5-Aza, six known bisabolane sesquiterpenes were harvested (Wang C.Y. et al., 2016). In present study, two new nucleoside derivatives (**1** and **2**), together with four known analogs were discovered by adding SAHA and 5-Aza simultaneously, which was confirmed by HPLC detection (Figure 1) and molecular networking analysis (Figure 3). Compounds **1** and **2** were newly emerged metabolites in the fungal culture relative to control (Figure 1 and Supplementary Figure S30). Quantification analysis revealed that compounds **3** and **4** from the culture with a combination of SAHA and 5-Aza were upregulated approximately 365 fold and 81 fold, respectively, compared to those of control (Supplementary Figures S29, S30 and Supplementary Table S1). In the enlarged cluster of molecular networking, the newly emerged node, belonging to nucleoside derivatives could only be observed in the extracts of culture with the presence of 5-Aza (Figure 3). In addition, eleven bisabolane sesquiterpenes including one new compound were mined, which further confirmed the effect of chemical epigenetic manipulation on this strain to produce bisabolane sesquiterpenes.

### Bioassays of Compounds

The aroyl uridine derivatives have been reported to possess potential antibacterial activities. For example, kipukasins A and B, obtained from *A. versicolor* NRRL 35600 showed antibacterial activities against Gram-positive bacteria (Jiao et al., 2007). Chen et al. (2014a) reported the uridine derivatives kipukasins H/I exhibited antibacterial activity against *S. epidermidis*. For bisabolane sesquiterpenes, multiple activities have been reported, such as antimicrobial (Li et al., 2012; Guo et al., 2018), antiviral (Wang et al., 2014), and antifouling (Li et al., 2012) activities. In this study, all of the isolated compounds were evaluated for their antibacterial, antifungal, cytotoxic, and antifouling activities. Compounds **1** and **7** showed antibacterial activities against *S. epidermidis* and *S. aureus* with the MIC values of 8–16 μg/mL, respectively. When the hydroxyl at C-4″ in compounds **1**, **3,** and **4** was replaced by methoxyl in **2**, **5,** and **6**, the antibacterial activities were significantly decreased, respectively. While the hydroxyl at C-5′ in **3** and **4** was substituted by protocatechuic ester in **1** and **2**, no remarkable change was observed for the antibacterial activity. In addition, compound **7** exhibited antifungal activities against *Candida albicans* and *C. tropicalis* with the MIC values of 64 and 32 μg/mL, respectively. Unfortunately, none of the tested compounds showed cytotoxicity against human tumor cell lines A549, HCT116, MCF-7, Hela, and Hep G2. Interestingly, compound **7** exhibited anti-larval attachment activity against bryozoan *B. neritina* with the EC_50_ and LC_50_ values of 6.25 and 25 μg/mL, respectively. At the concentrations of 25 μg/mL, compounds **1**–**3**, **6**, and **14** could inhibit the larval settlement, while **8** could kill the larval.

## Conclusion

In summary, the gorgonian-derived fungus *A*. *versicolor* XS-20090066 was effectively induced by chemical epigenetic manipulation with a combination of 100 μM SAHA and 100 μM 5-Aza to produce two new nucleoside derivatives and one new bisabolane sesquiterpene as well as more known derivatives. These induced metabolites exhibited antibacterial, antifungal and antifouling activities. Therefore, it could be concluded that chemical epigenetic manipulation should be a feasible and effective strategy to trigger the production of bioactive secondary metabolites from marine derived-fungi.

## Data Availability Statement

The datasets generated for this study can be found in the GenBank (NCBI) accession number MN880095.

## Author Contributions

C-YW and YL conceived and proposed the idea. J-SW contributed to the fermentation, extraction, isolation, and manuscript preparation. G-SY, X-HS, and YX contributed to the bioactivities test. J-SW, SR, X-MF, and X-LZ contributed to the data analysis, writing, revising, and proofreading of the manuscript. All authors read and approved the final version of the manuscript.

## Conflict of Interest

The authors declare that the research was conducted in the absence of any commercial or financial relationships that could be construed as a potential conflict of interest.
