# Supplementary material for: Epigenetic Agents Trigger the Production of Bioactive Nucleoside Derivatives and Bisabolane Sesquiterpenes From the Marine-Derived Fungus Aspergillus versicolor
Source: Front Microbiol. 2020 Jan 30;11:85. doi: 10.3389/fmicb.2020.00085 (PMC7002437; doi:10.3389/fmicb.2020.00085)
Supplement: Supplementary file 1 [file Data_Sheet_1.PDF]

Epigenetic agents trigger the production of bioactive nucleoside derivatives and bisabolane sesquiterpenes from the marine-derived fungus *Aspergillus versicolor*

Jing-Shuai Wu [1,2], Guang-Shan Yao [1,2,3], Xiao-Hui Shi [1,2], Saif Ur Rehman [1,2], Ying Xu [4], Xiu-Mei Fu [1,2], Xiu-Li Zhang [1,2], Yang Liu [5]* and Chang-Yun Wang [1,2]*

[1] Key Laboratory of Marine Drugs, The Ministry of Education of China, School of Medicine and Pharmacy, Institute of Evolution & Marine Biodiversity, Ocean University of China, Qingdao 266003, People's Republic of China
[2] Laboratory for Marine Drugs and Bioproducts, Qingdao National Laboratory for Marine Science and Technology, Qingdao 266237, People's Republic of China
[3] Institute of Oceanography, Minjiang University, Fuzhou 350108, People's Republic of China
[4] Shenzhen Key Laboratory of Marine Bioresource & Eco-Environmental Science, Shenzhen Engineering Laboratory for Marine Algal Biotechnology, College of Life Sciences and Oceanography, Shenzhen University, Shenzhen 518060, People's Republic of China
[5] Institute for Insect Biotechnology, Department of Bioresources of the Fraunhofer Institute for Molecular Biology and Applied Ecology (IME), Justus-Liebig-University of Giessen, 35392, Giessen, Germany

*** Correspondence:**

Chang-Yun Wang
changyun@ouc.edu.cn

Yang Liu
Liu.Yang@agrar.uni-giessen.de

List of Supporting Information

Figure S1. The phylogenetic tree of fungal strain

Figure S2. The schematic diagram of separation of compounds

Figure S3. ^{1}H NMR (600 MHz, DMSO) spectrum of compound **1**

Figure S4. ^{13}C NMR (150 MHz, DMSO) spectrum of compound **1**

Figure S5. HSQC (DMSO) spectrum of compound **1**

Figure S6. ^{1}H-^{1}H COSY (DMSO) spectrum of compound **1**

Figure S7. HMBC (DMSO) spectrum of compound **1**

Figure S8. ROESY (DMSO) spectrum of compound **1**

Figure S9. NOESY (DMSO) spectrum of compound **1**

Figure S10. ESIMS spectrum of compound **1**

Figure S11. HRESIMS spectrum of compound **1**

Figure S12. ^{1}H NMR (600 MHz, DMSO) spectrum of compound **2**

Figure S13. ^{13}C NMR (150 MHz, DMSO) spectrum of compound **2**

Figure S14. HSQC (DMSO) spectrum of compound **2**

Figure S15. ^{1}H-^{1}H COSY (DMSO) spectrum of compound **2**

Figure S16. HMBC (DMSO) spectrum of compound **2**

Figure S17. ROESY (DMSO) spectrum of compound **2**

Figure S18. NOESY (DMSO) spectrum of compound **2**

Figure S19. ESIMS spectrum of compound **2**

Figure S20. HRESIMS spectrum of compound **2**

Figure S21. ROESY correlations of compounds **1** and **2**

Figure S22. ^{1}H NMR (500 MHz, DMSO) spectrum of compound **7**

Figure S23. ^{13}C NMR (125 MHz, DMSO) spectrum of compound **7**

Figure S24. HSQC (DMSO) spectrum of compound **7**

Figure S25. ^{1}H-^{1}H COSY (DMSO) spectrum of compound **7**

Figure S26. HMBC (DMSO) spectrum of compound **7**

Figure S27. ESIMS spectrum of compound **7**

Figure S28. HRESIMS spectrum of compound **7**

Figure S29. The standard curves and the standard equations of compounds **1−4**

Figure S30. HPLC profiles containing compounds **1−4** in extracts

Table S1 The contents of compounds **1−4** in extracts of fungal strain

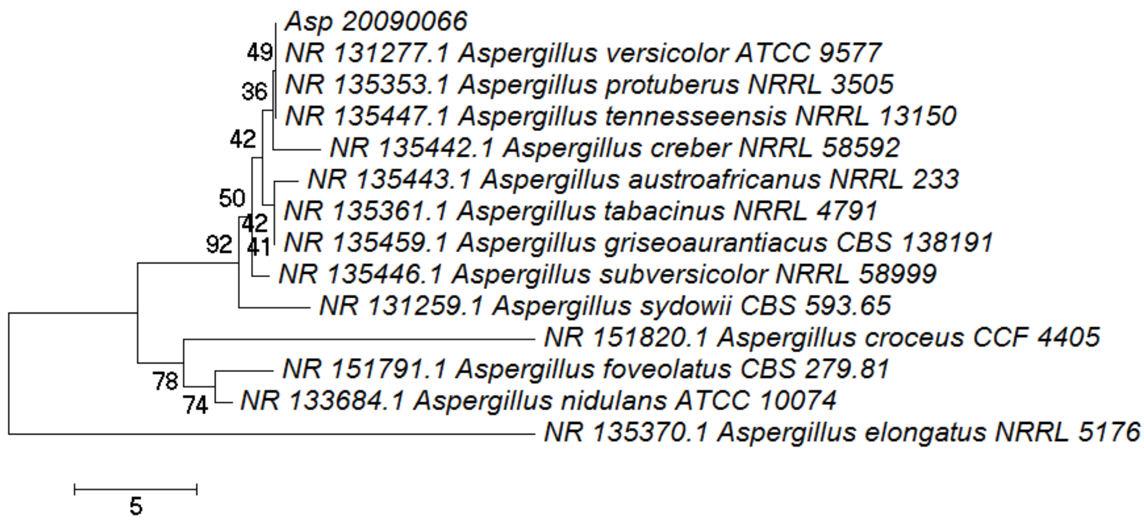

Figure S1. The phylogenetic tree of fungal strain

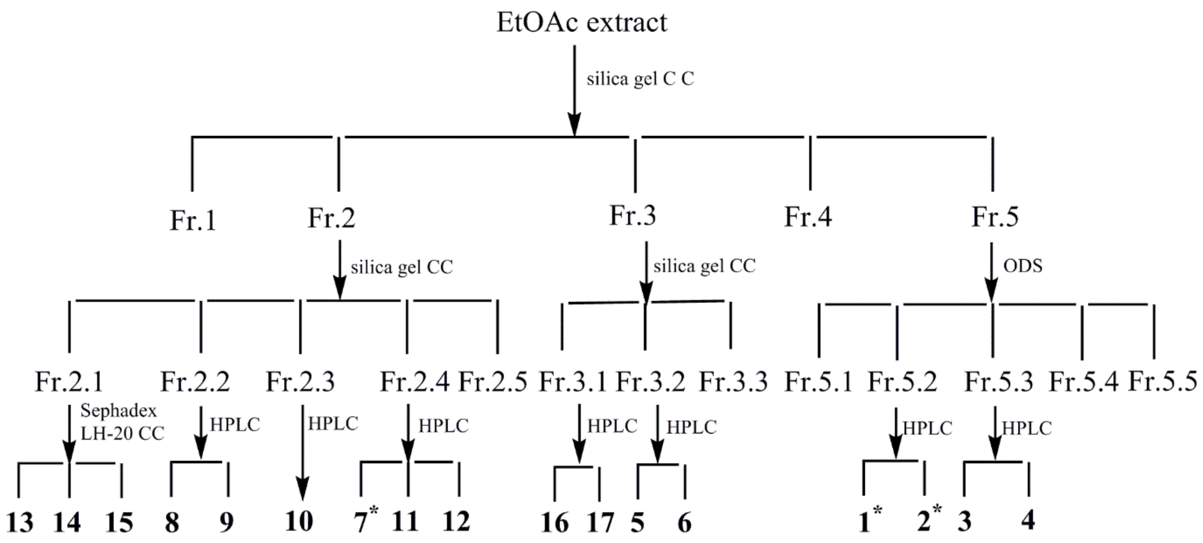

Figure S2. The schematic diagram of separation of compounds
(* represented the new compounds)

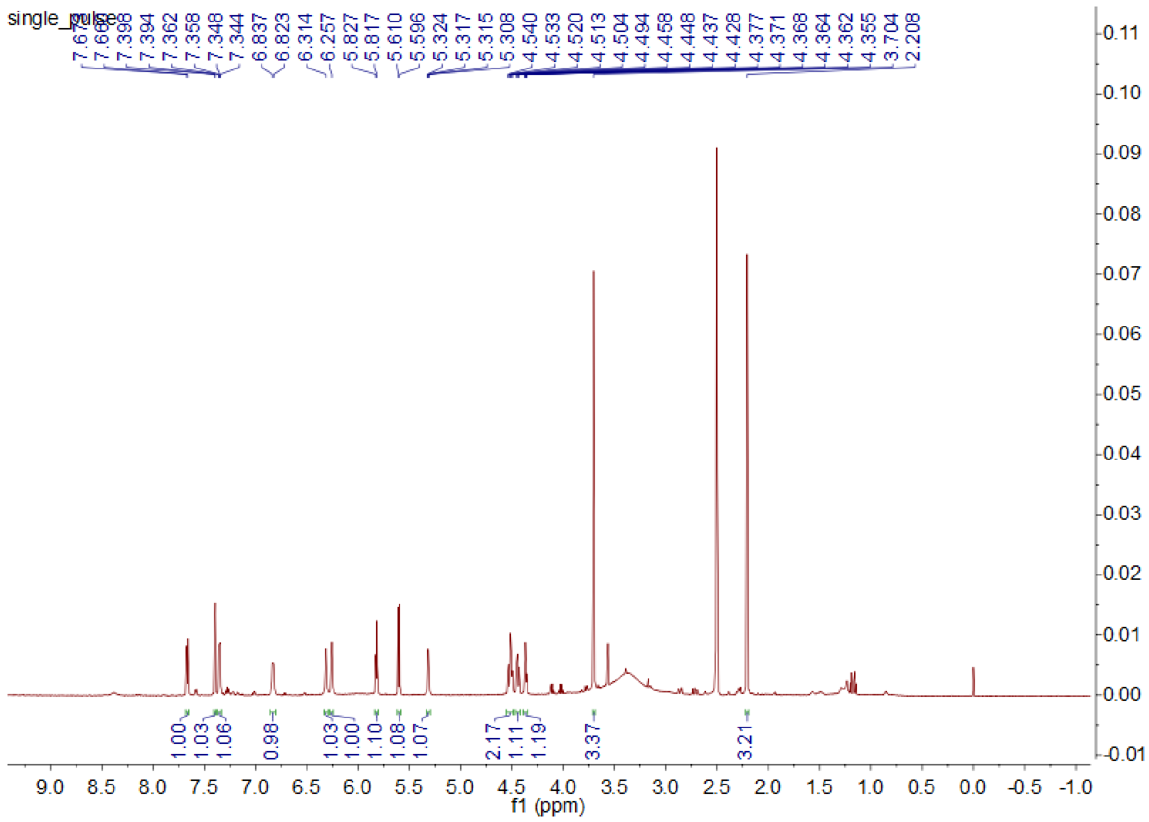

Figure S3. ^{1}H NMR (600 MHz, DMSO) spectrum of compound **1**

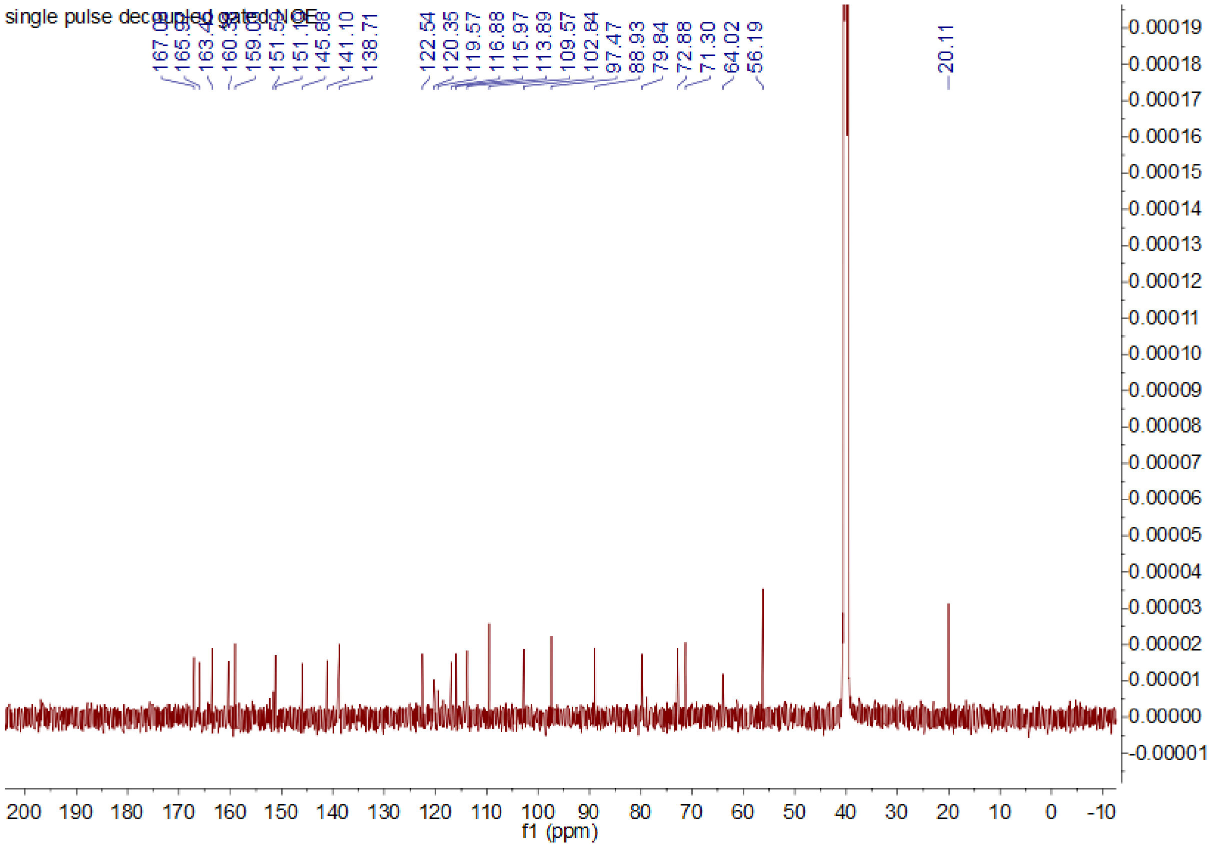

Figure S4. ^{13}C NMR (150 MHz, DMSO) spectrum of compound **1**

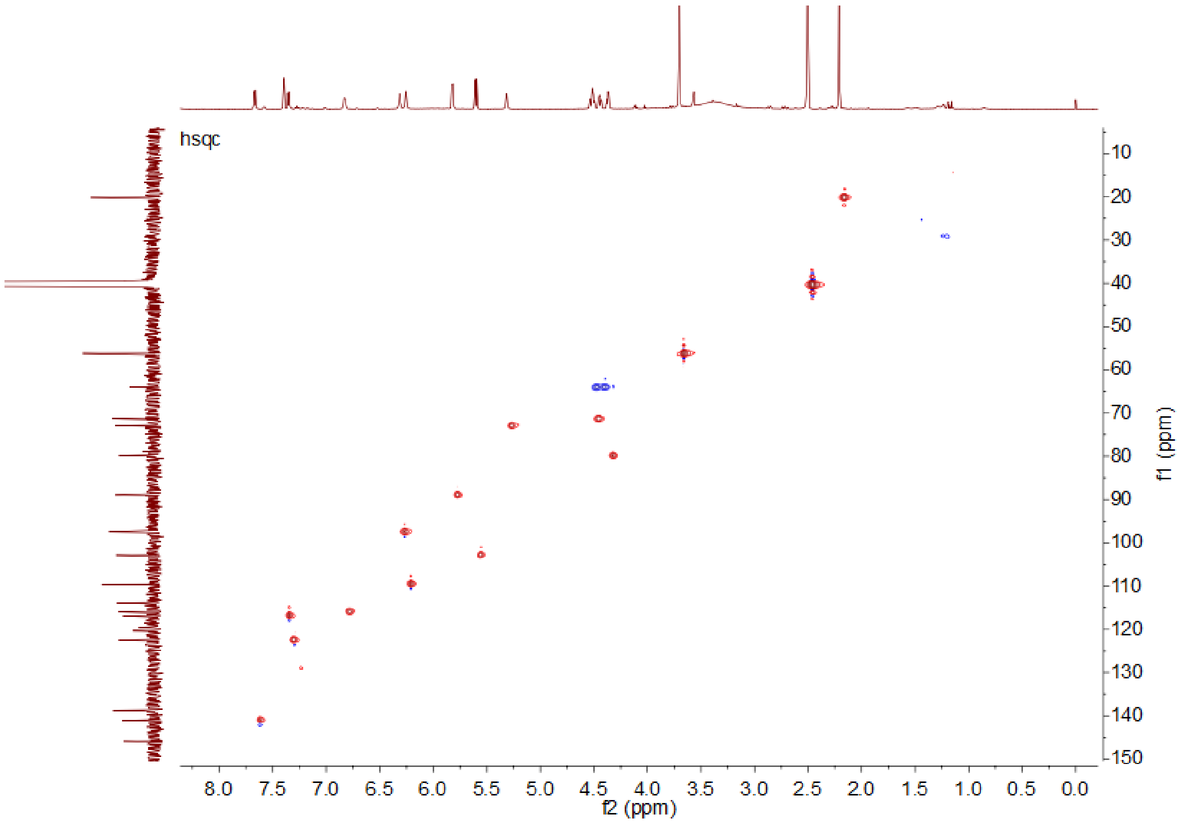

Figure S5. HSQC (DMSO) spectrum of compound **1**

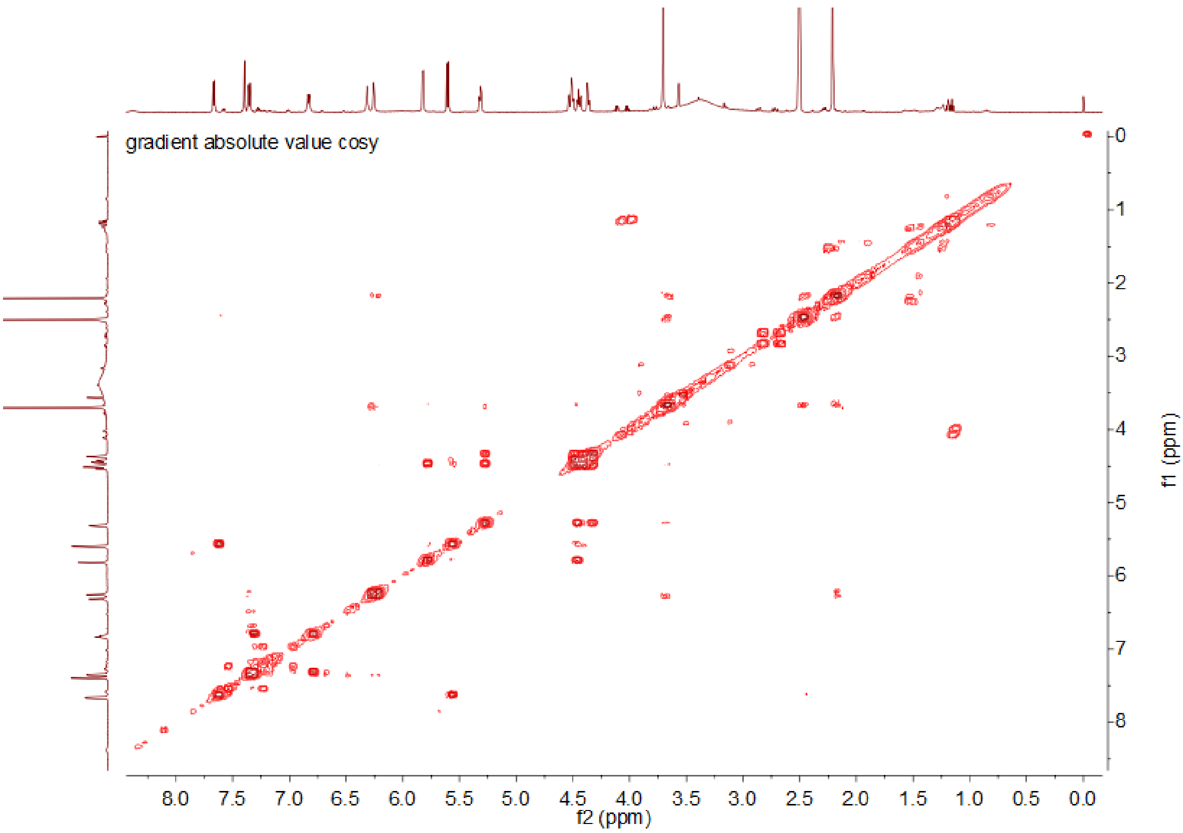

Figure S6. ^{1}H-^{1}H COSY (DMSO) spectrum of compound **1**

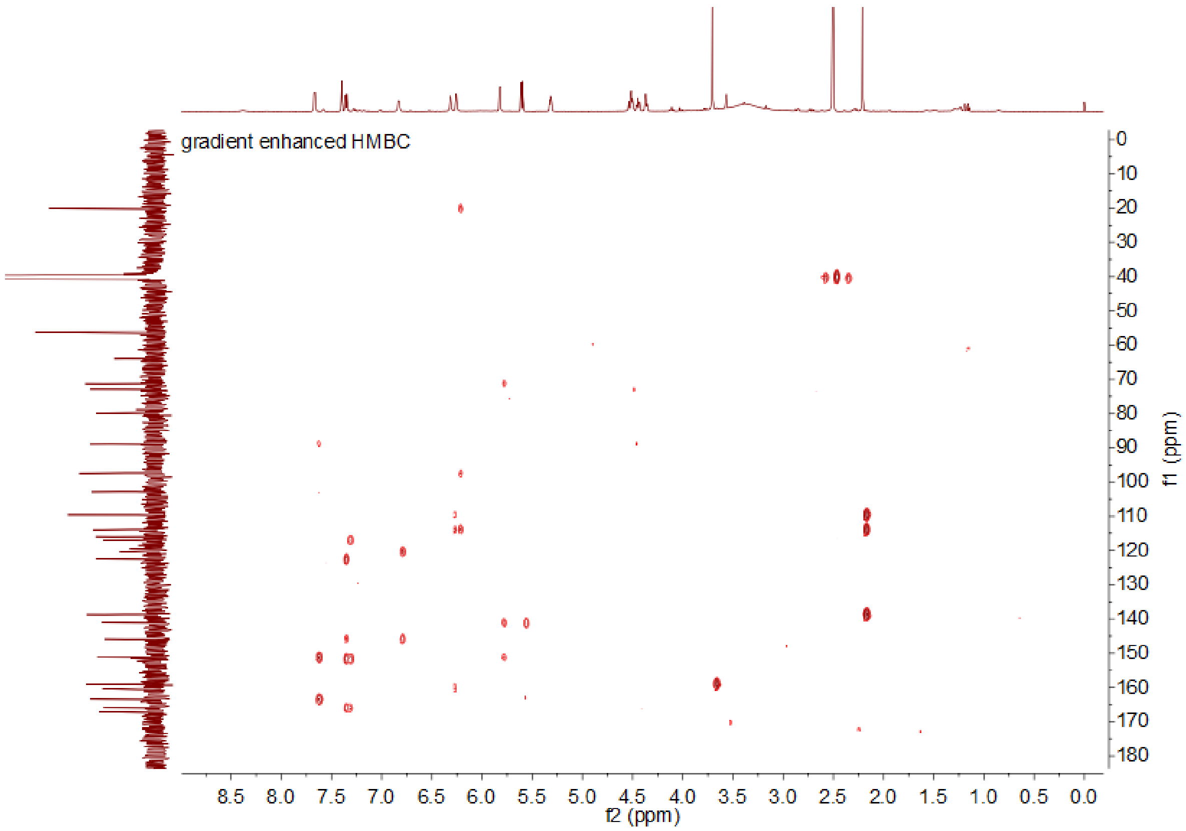

Figure S7. HMBC (DMSO) spectrum of compound **1**

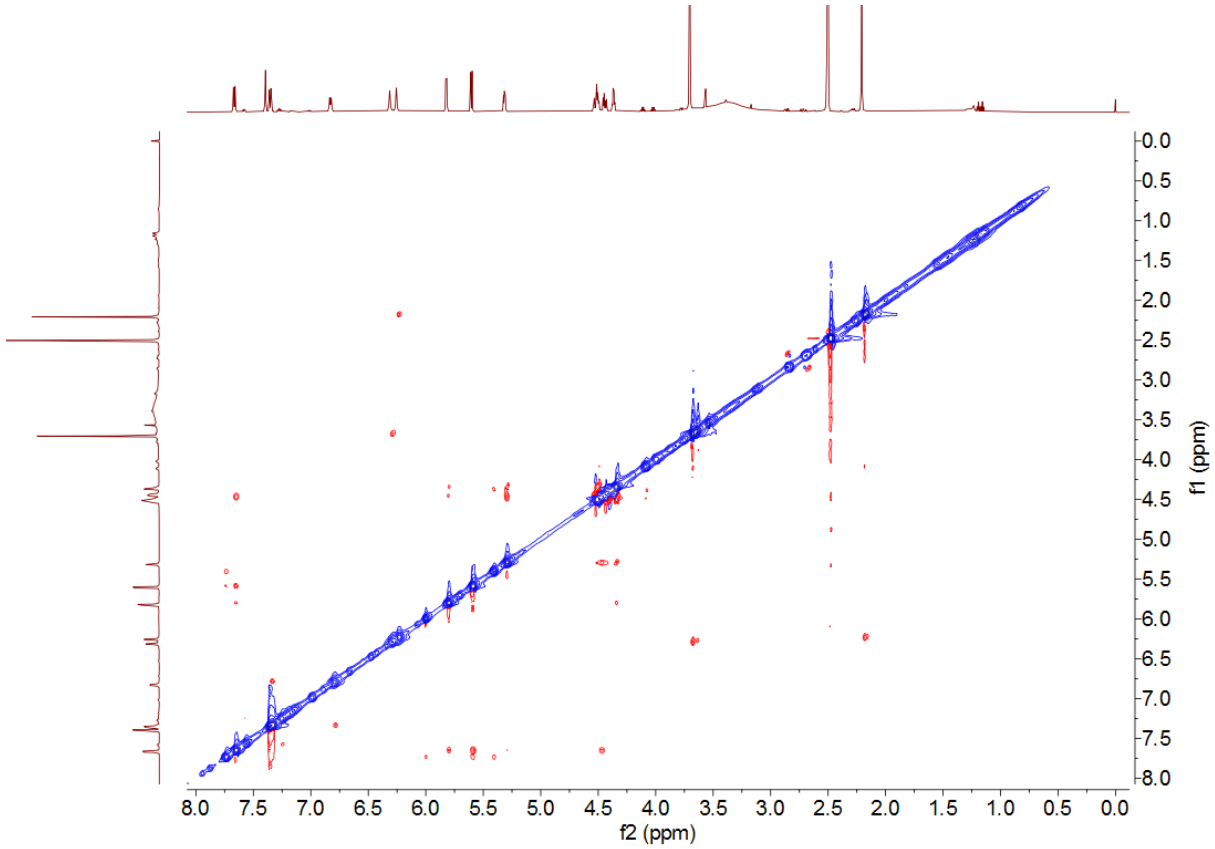

Figure S8. ROESY (DMSO) spectrum of compound **1**

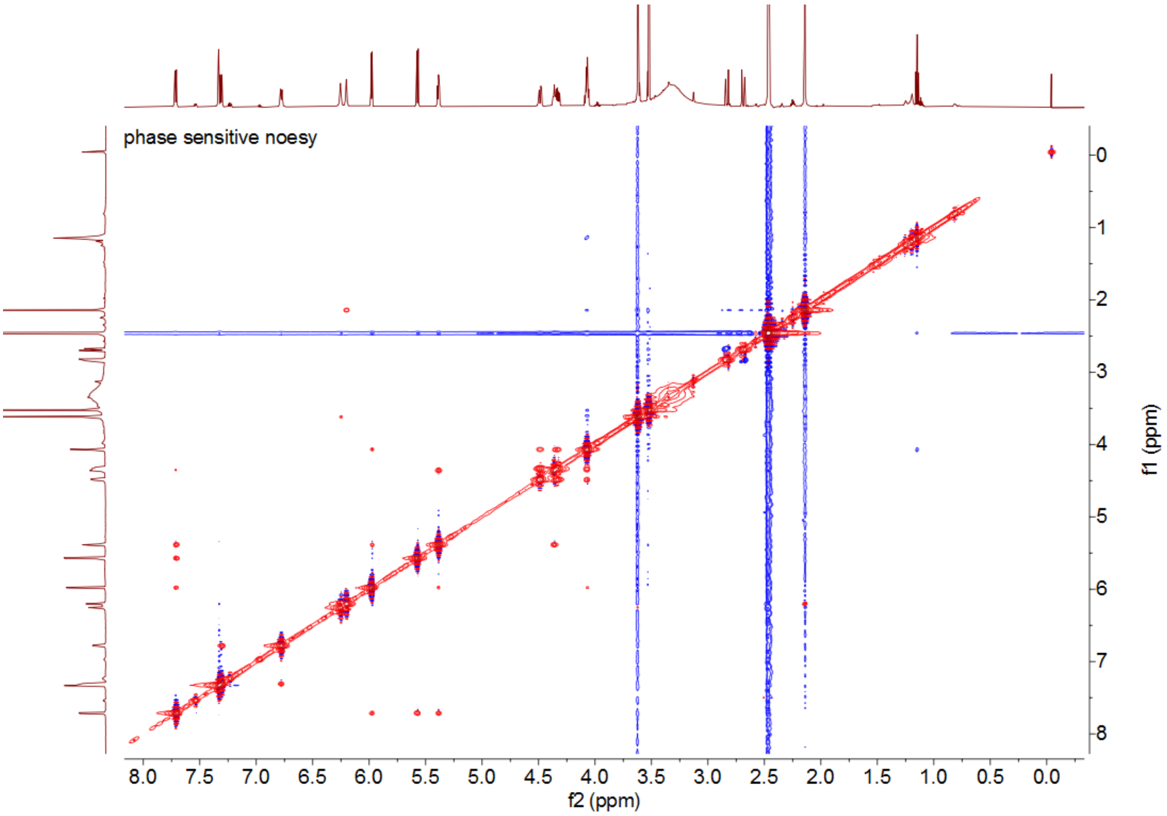

Figure S9. NOESY (DMSO) spectrum of compound **1**

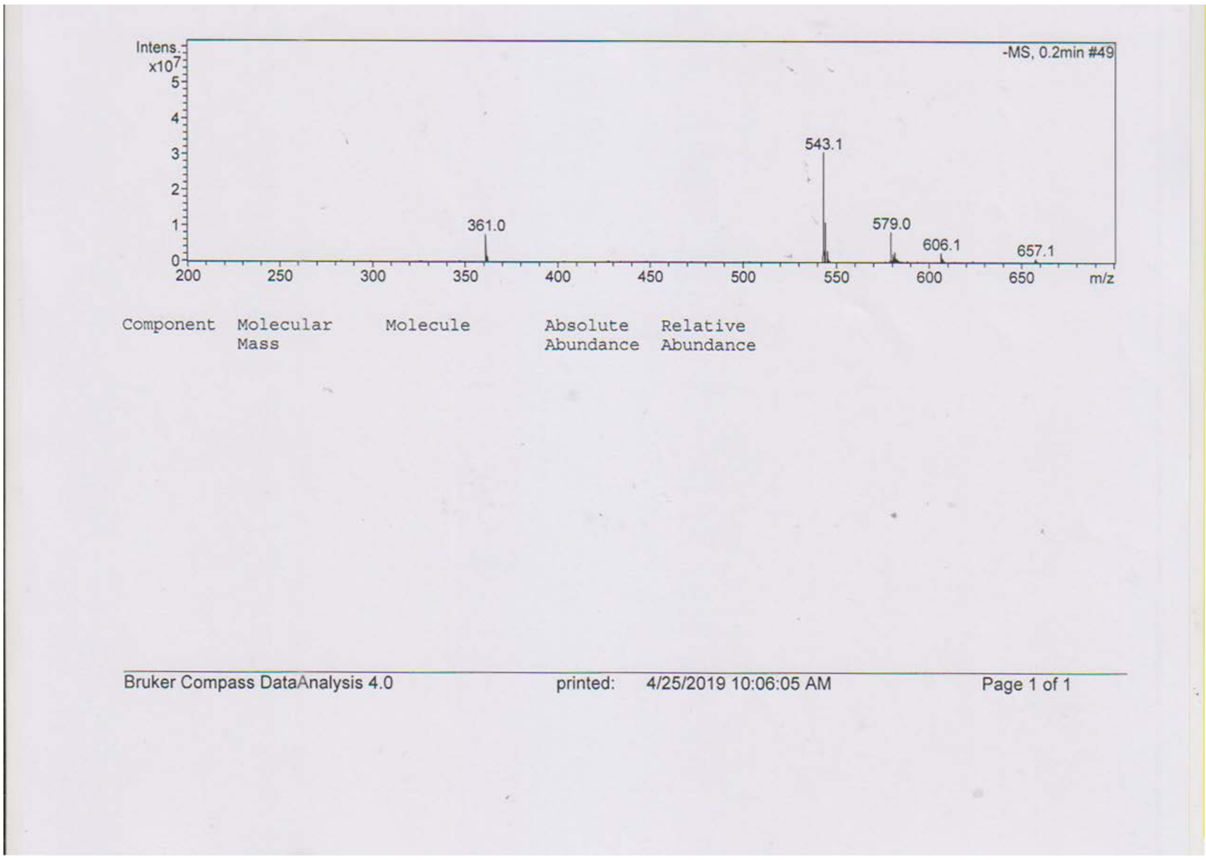

Figure S10. ESIMS spectrum of compound **1**

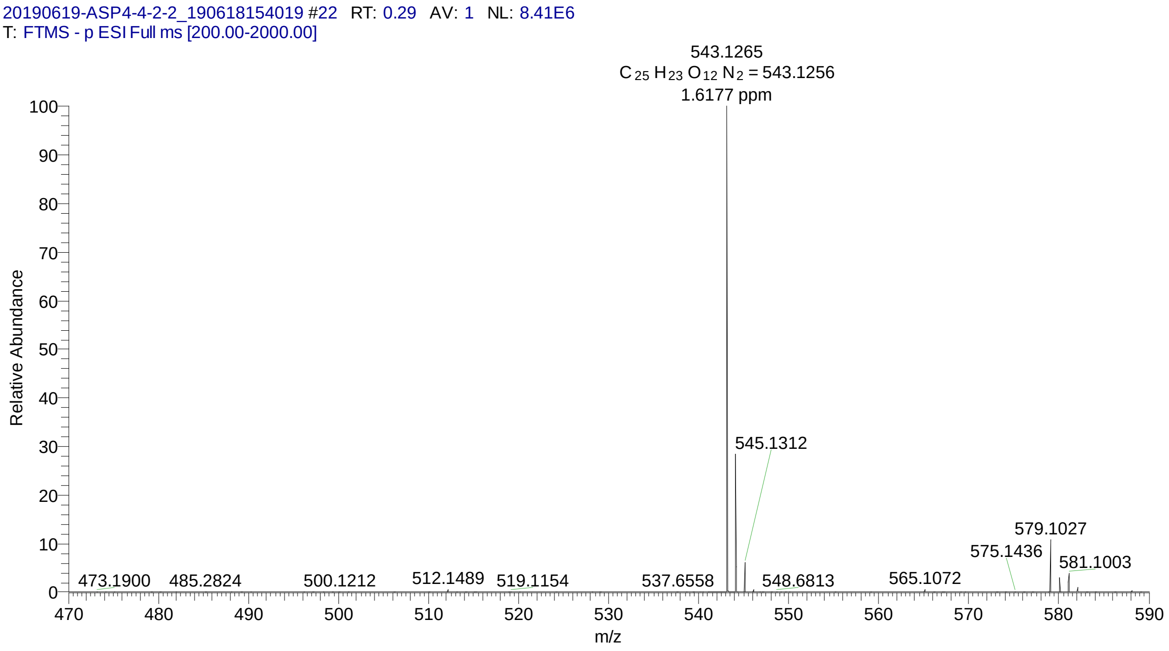

Figure S11. HRESIMS spectrum of compound **1**

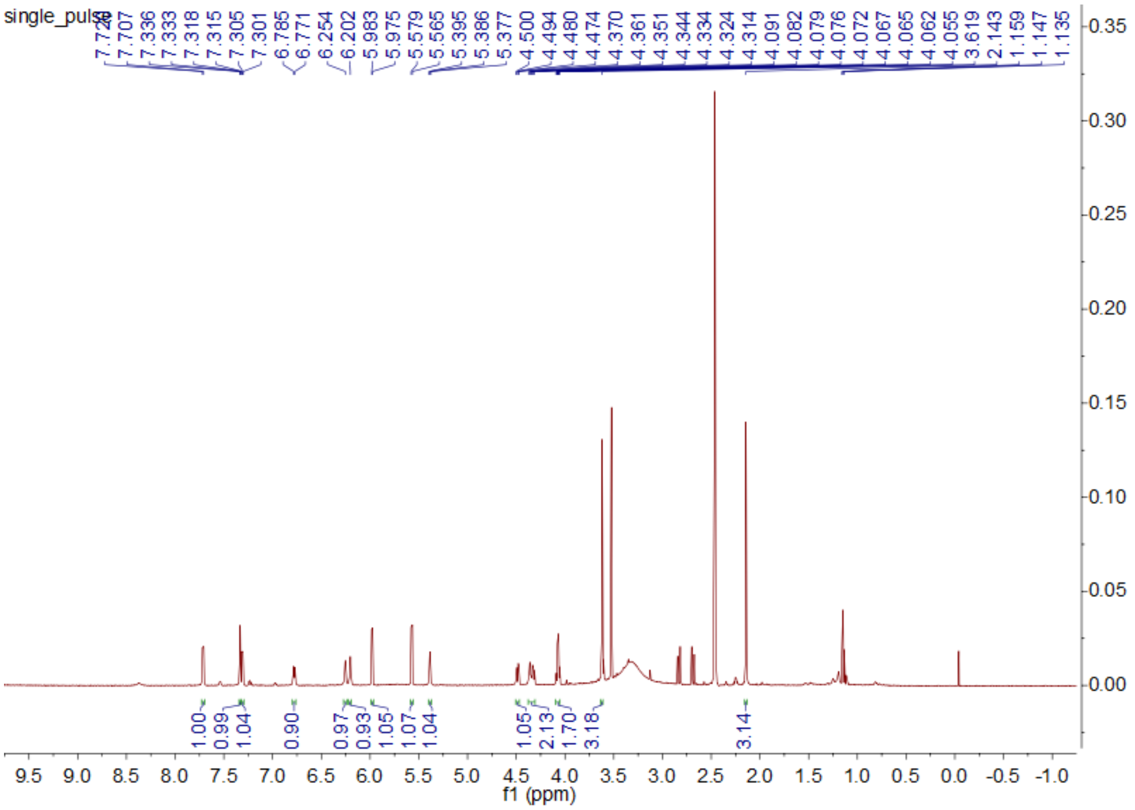

Figure S12. ^{1}H NMR (600 MHz, DMSO) spectrum of compound **2**

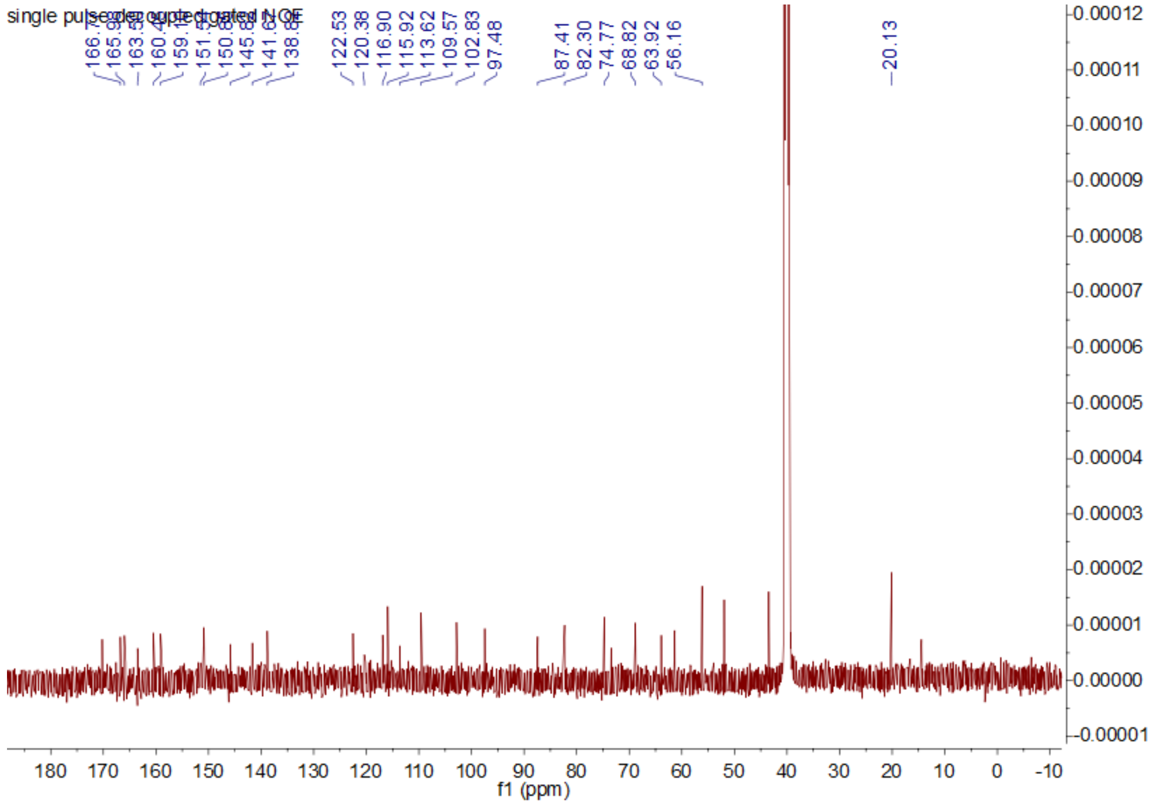

Figure S13. ^{13}C NMR (150 MHz, DMSO) spectrum of compound **2**

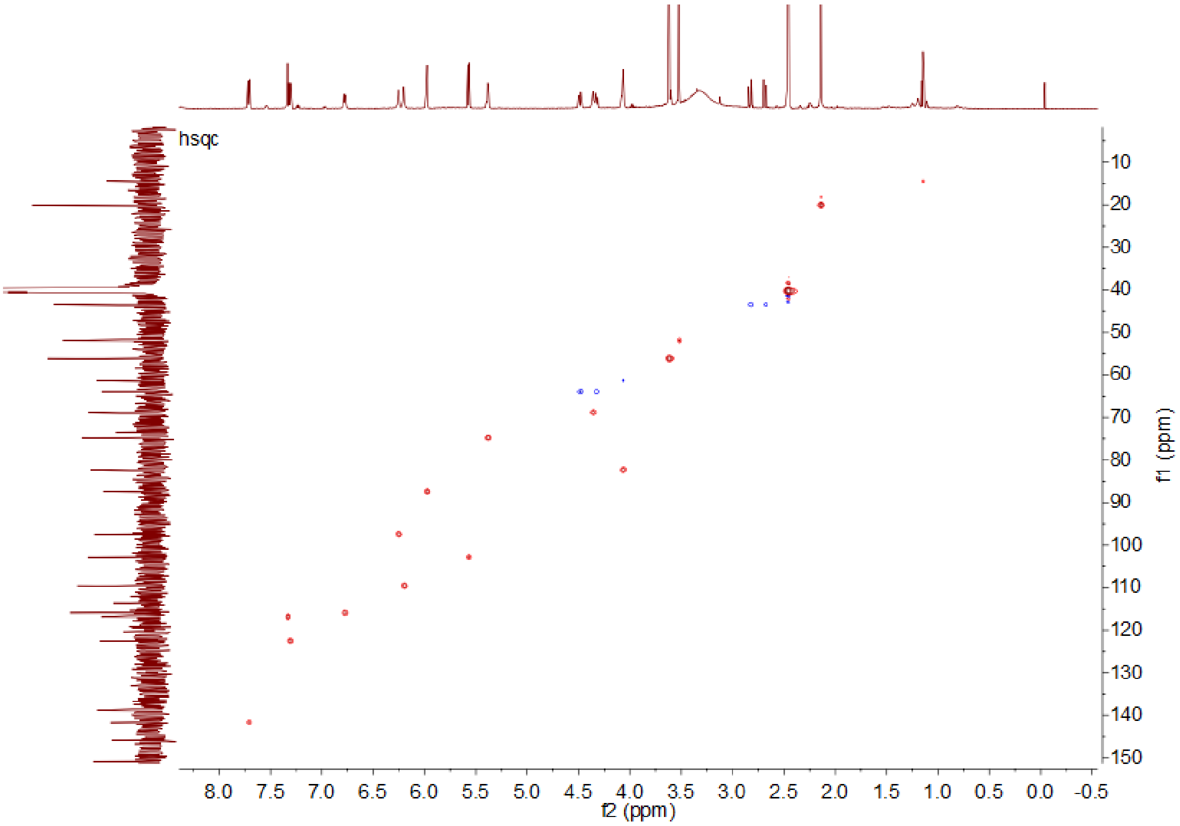

Figure S14. HSQC (DMSO) spectrum of compound **2**

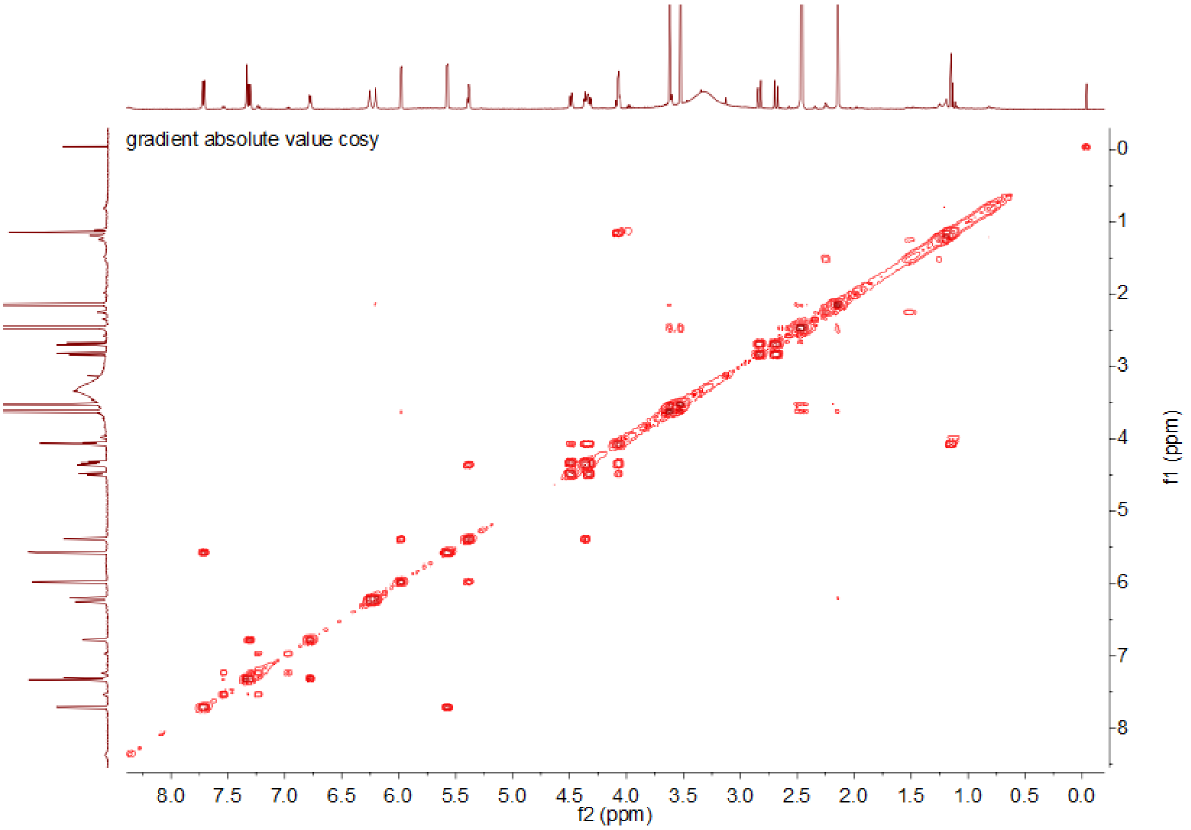

Figure S15. ^{1}H-^{1}H COSY (DMSO) spectrum of compound **2**

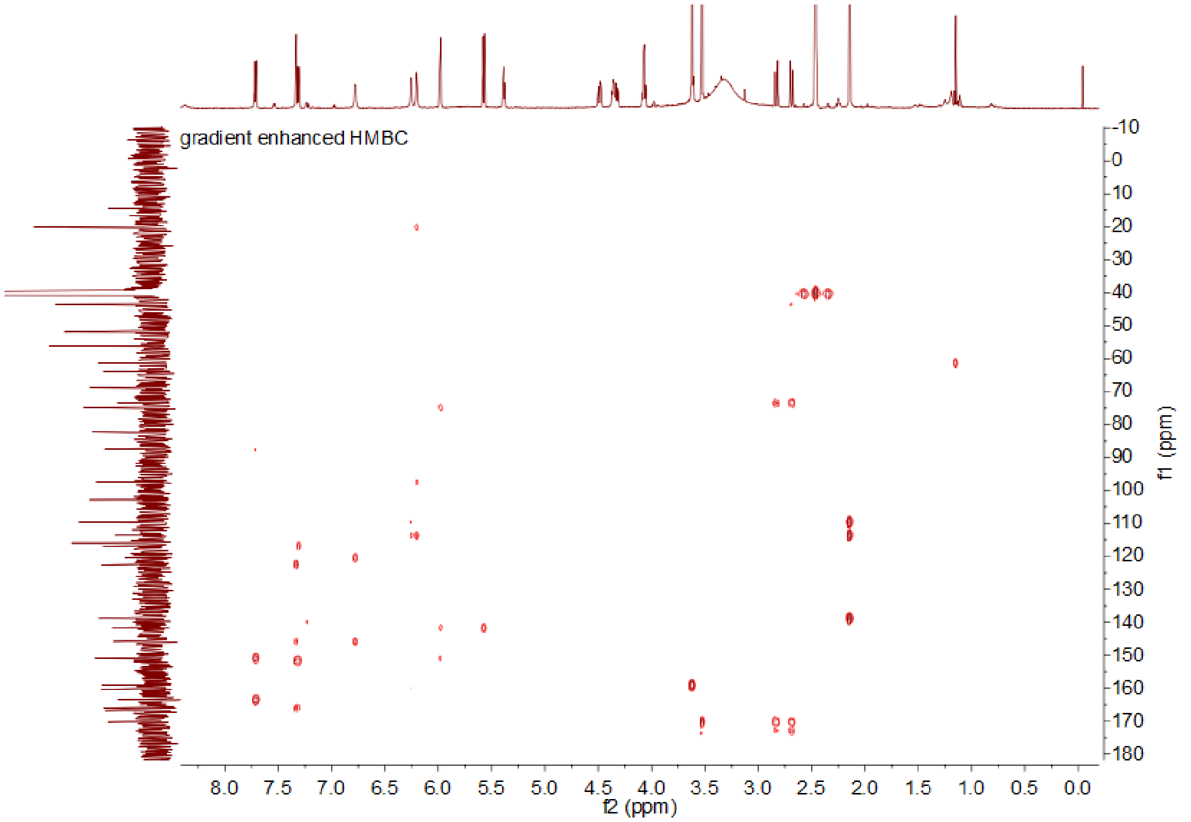

Figure S16. HMBC (DMSO) spectrum of compound **2**

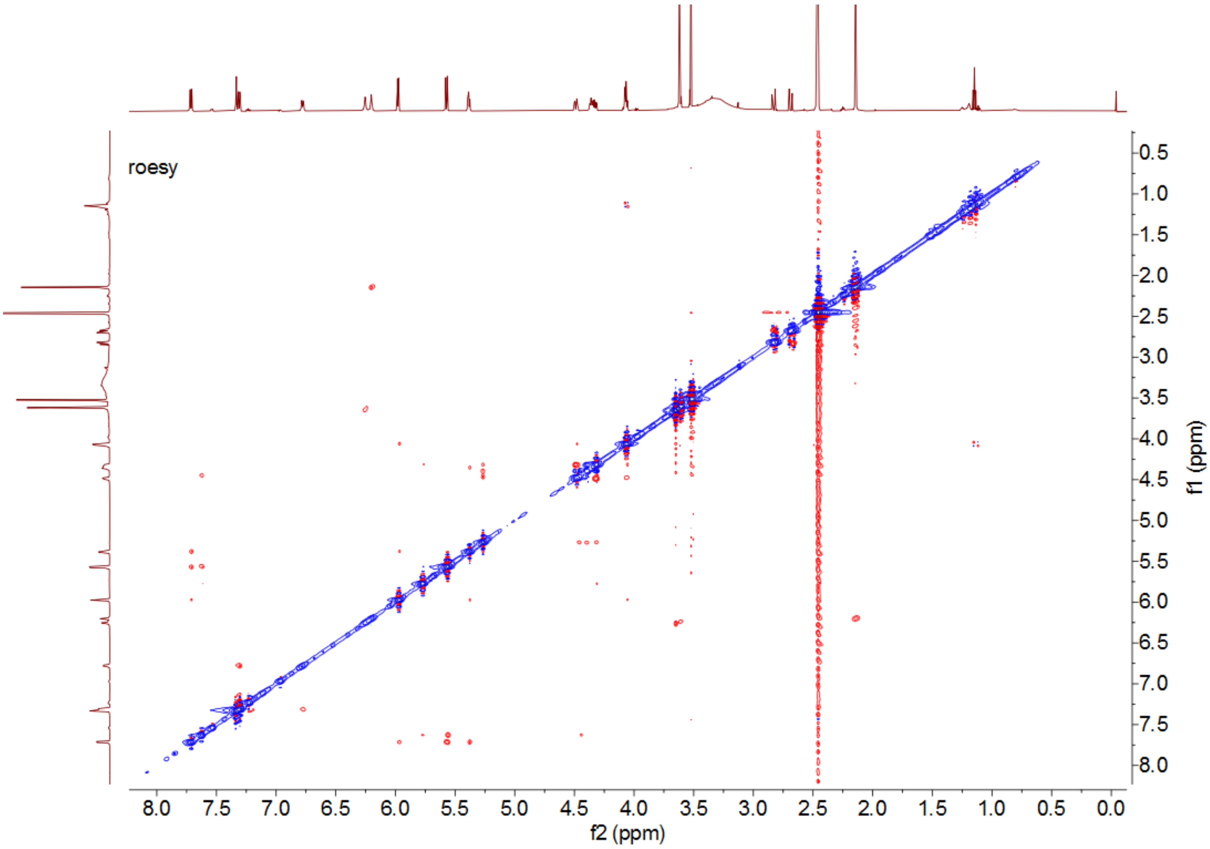

Figure S17. ROESY (DMSO) spectrum of compound **2**

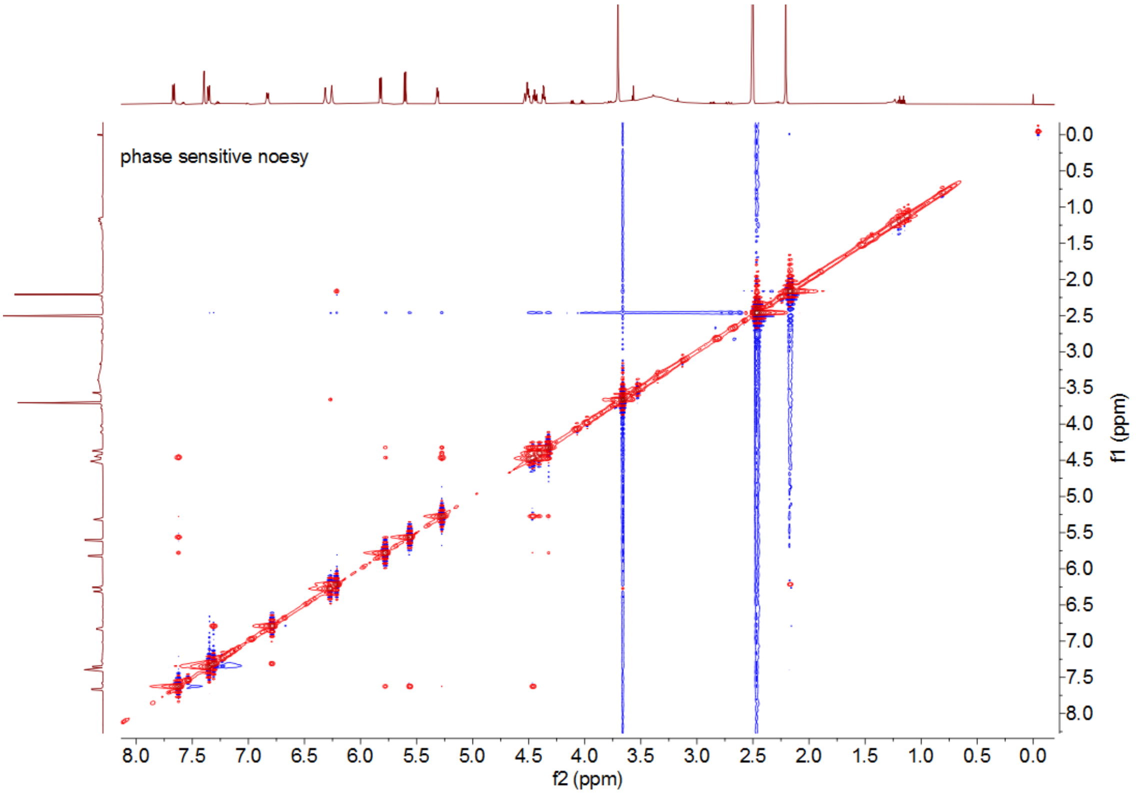

Figure S18. NOESY (DMSO) spectrum of compound **2**

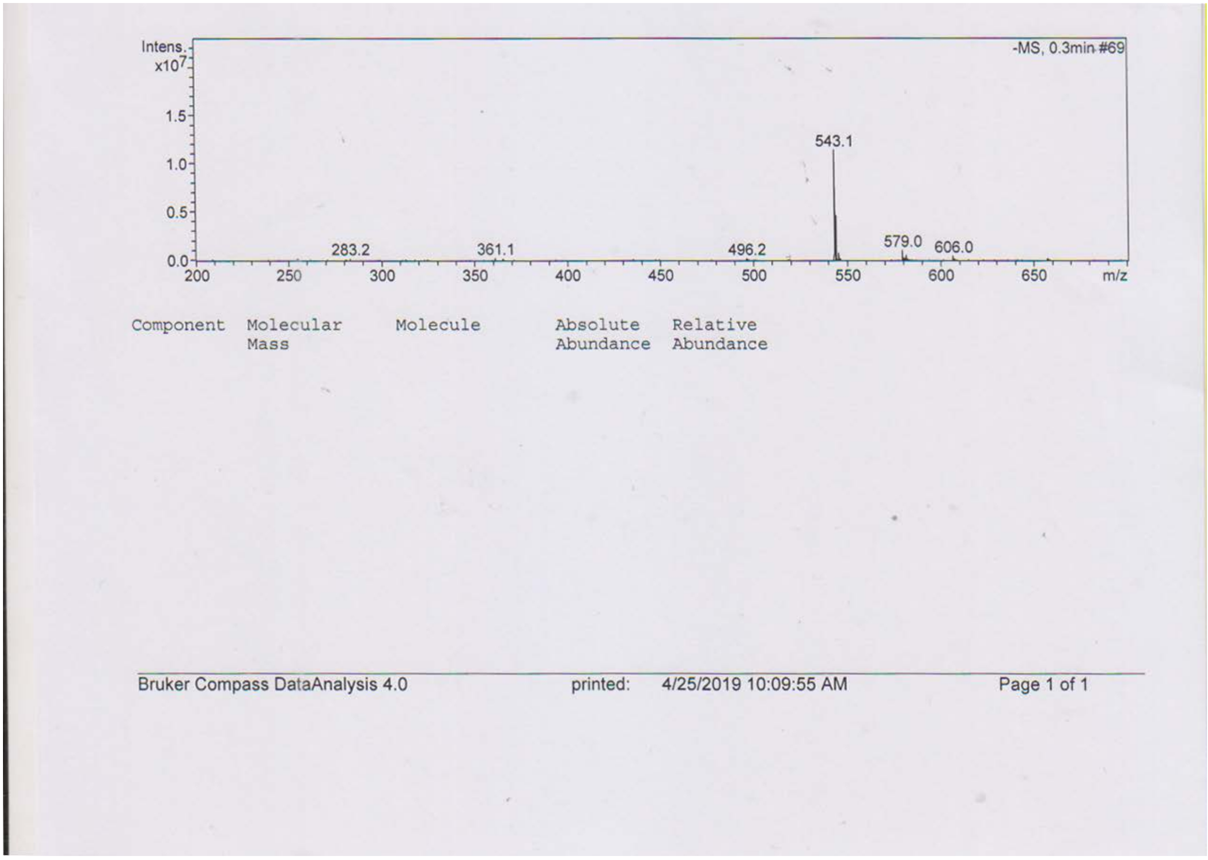

Figure S19. ESIMS spectrum of compound **2**

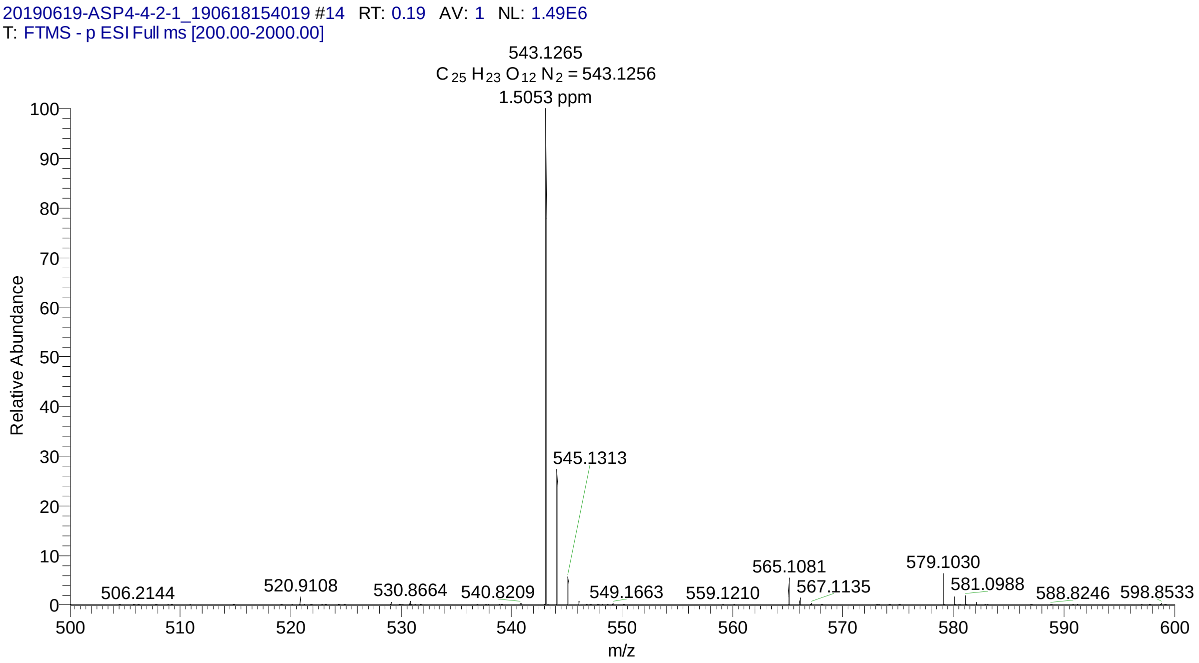

Figure S20. HRESIMS spectrum of compound **2**

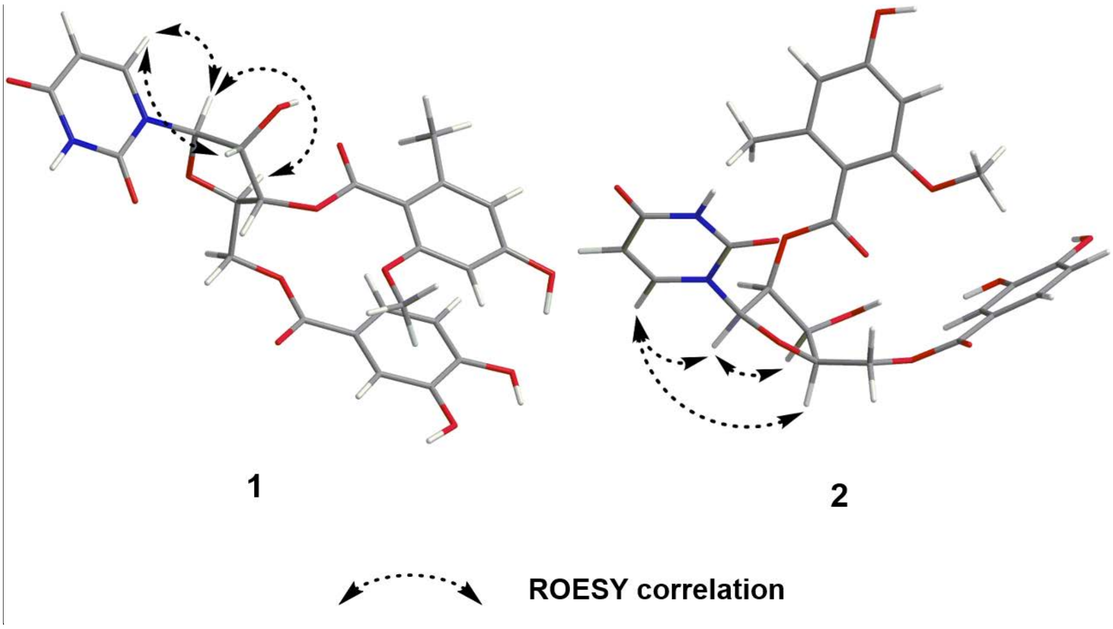

Figure S21. ROESY correlations of compounds **1** and **2**

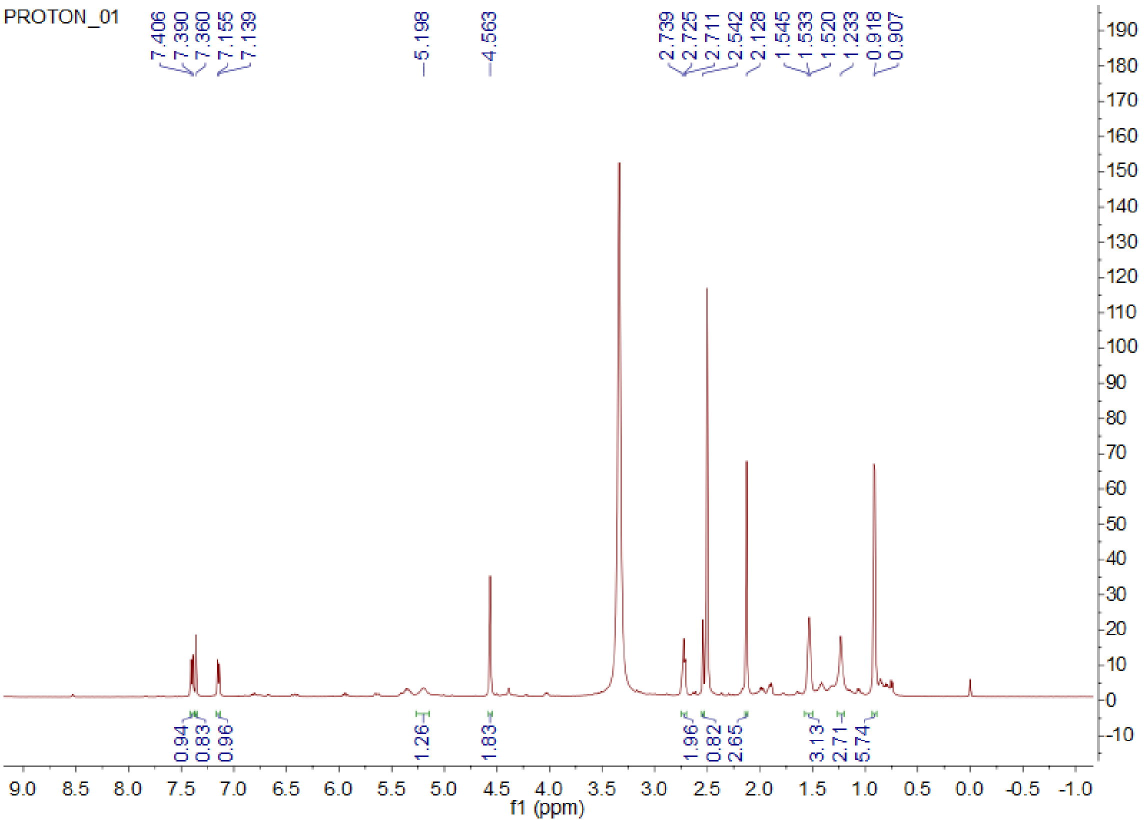

Figure S22. ^{1}H NMR (500 MHz, DMSO) spectrum of compound **7**

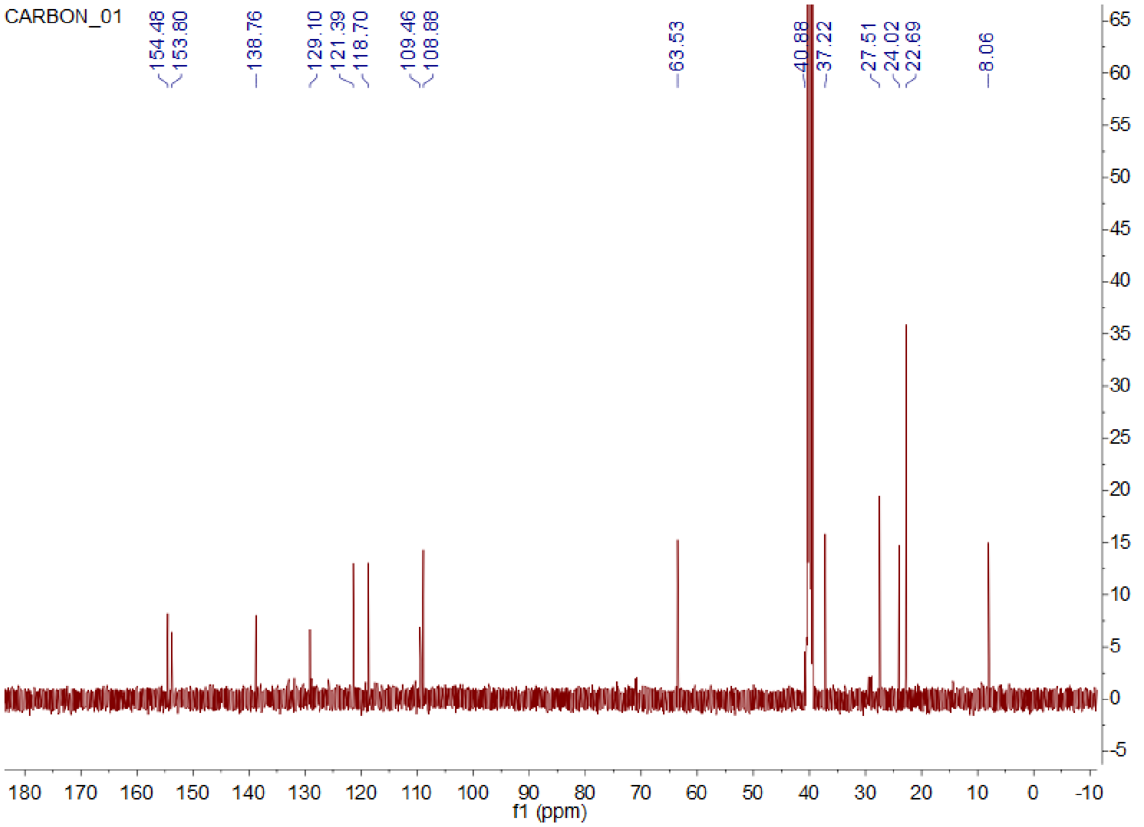

Figure S23. ^{13}C NMR (125 MHz, DMSO) spectrum of compound **7**

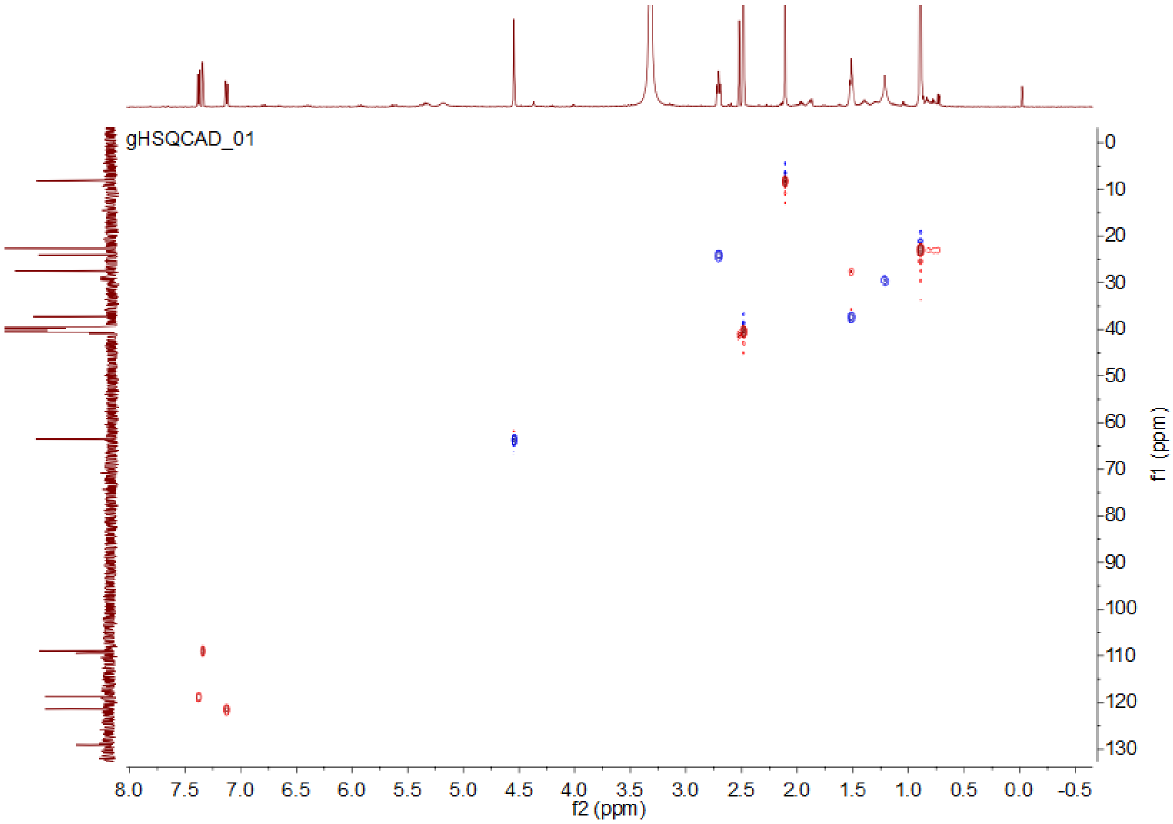

Figure S24. HSQC (DMSO) spectrum of compound **7**

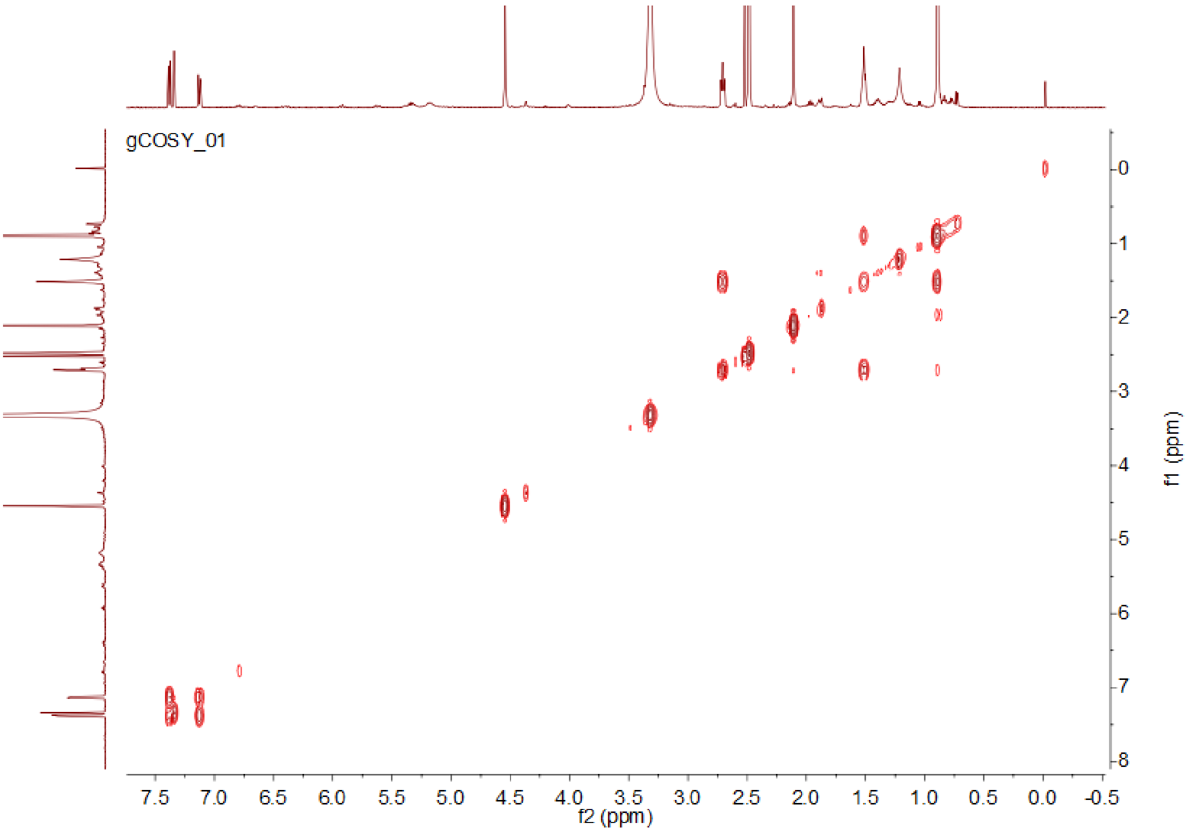

Figure S25. ^{1}H-^{1}H COSY (DMSO) spectrum of compound **7**

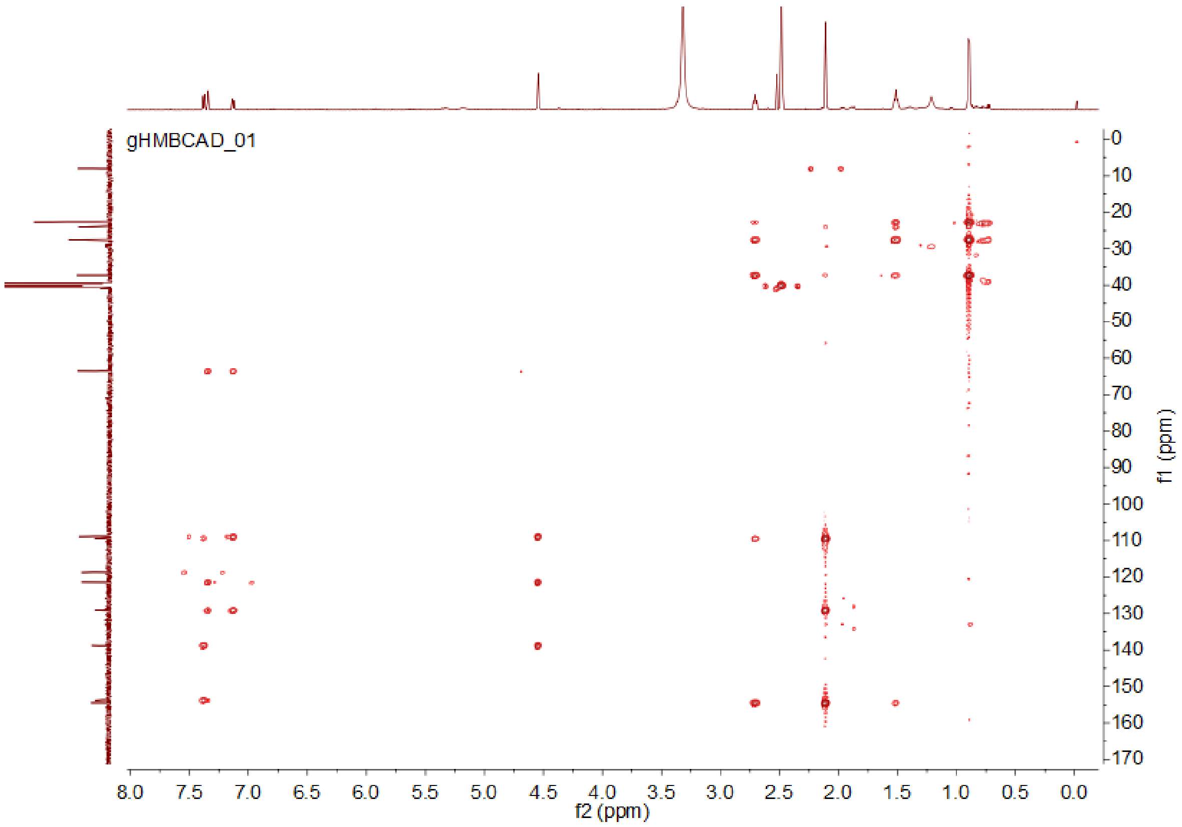

Figure S26. HMBC (DMSO) spectrum of compound **7**

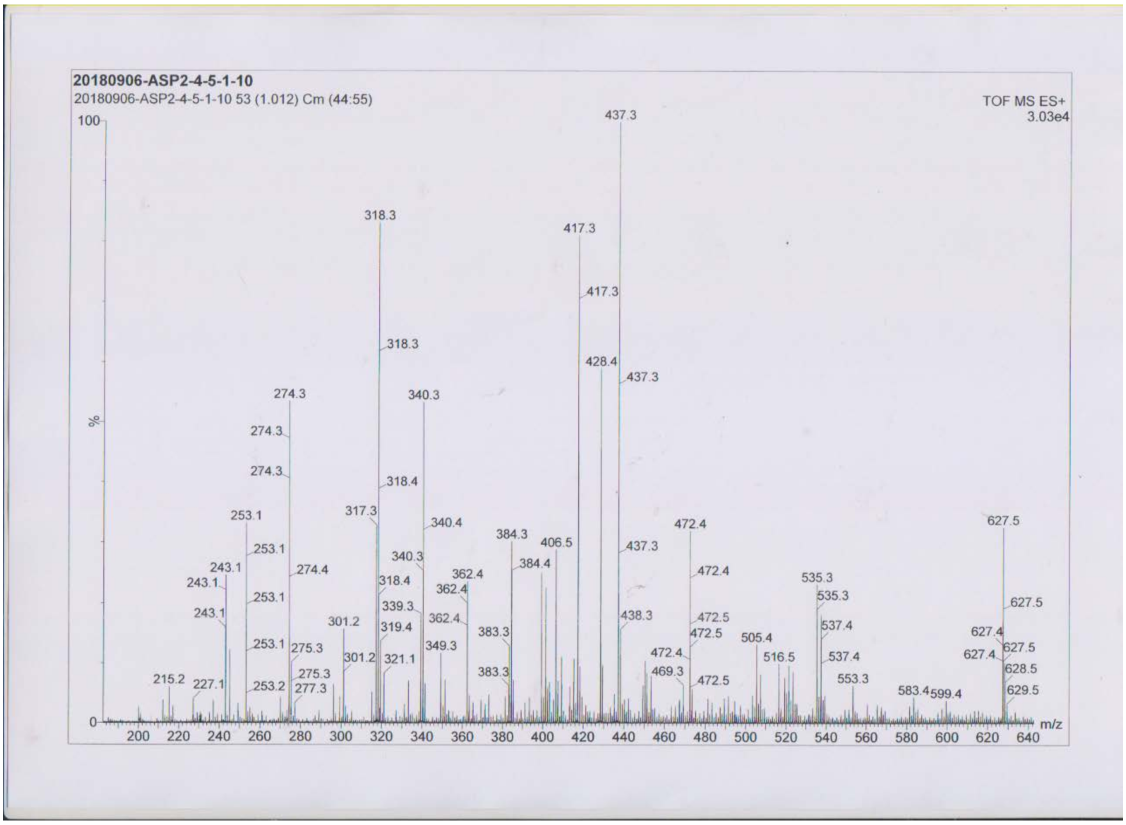

Figure S27. ESIMS spectrum of compound **7**

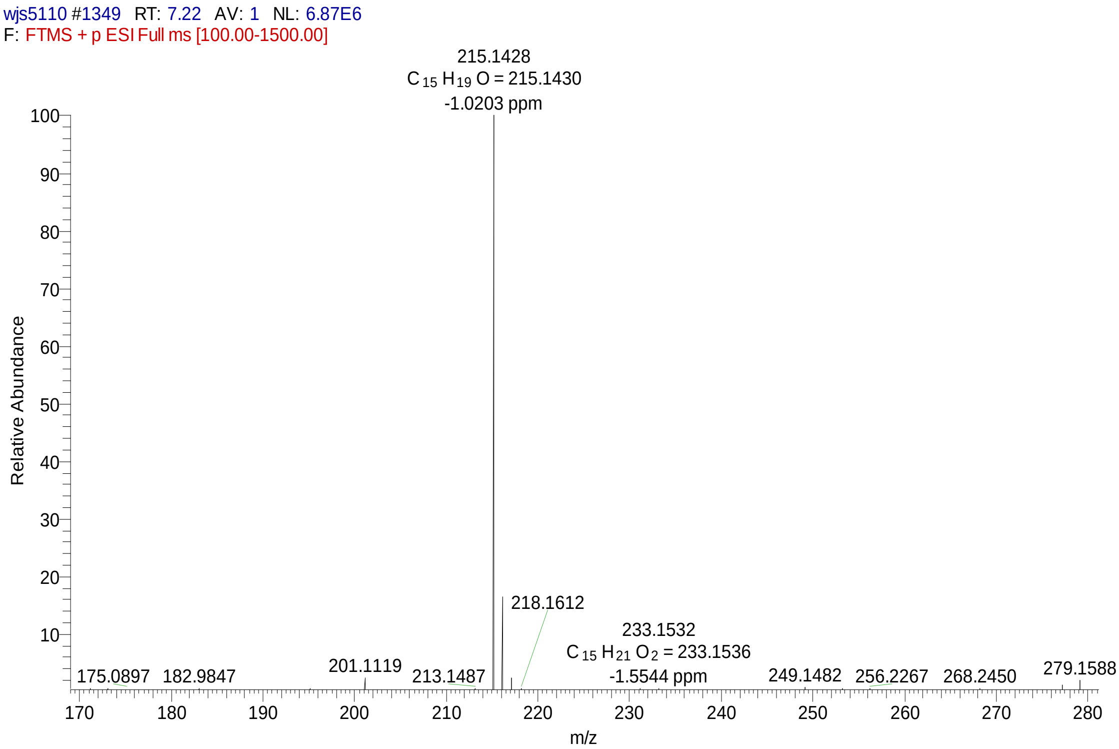

Figure S28. HRESIMS spectrum of compound **7**

.

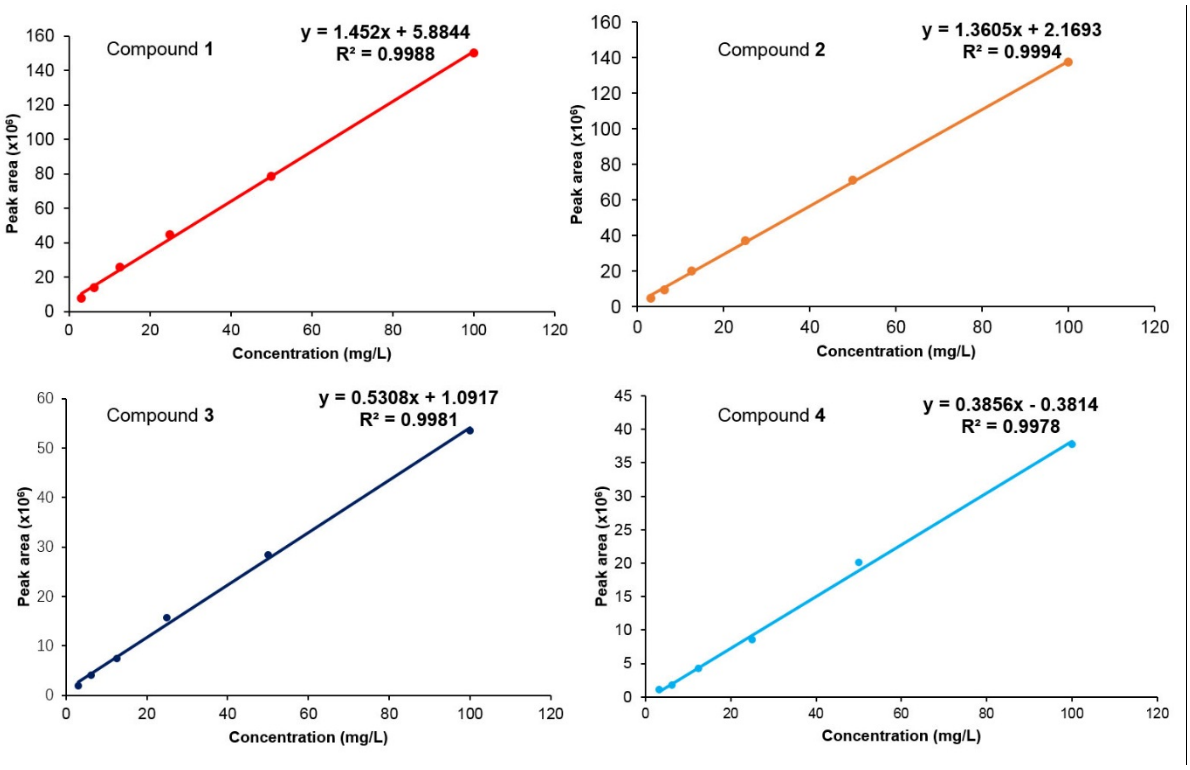

Figure S29. The standard curves and the standard equations of compounds **1**−**4**

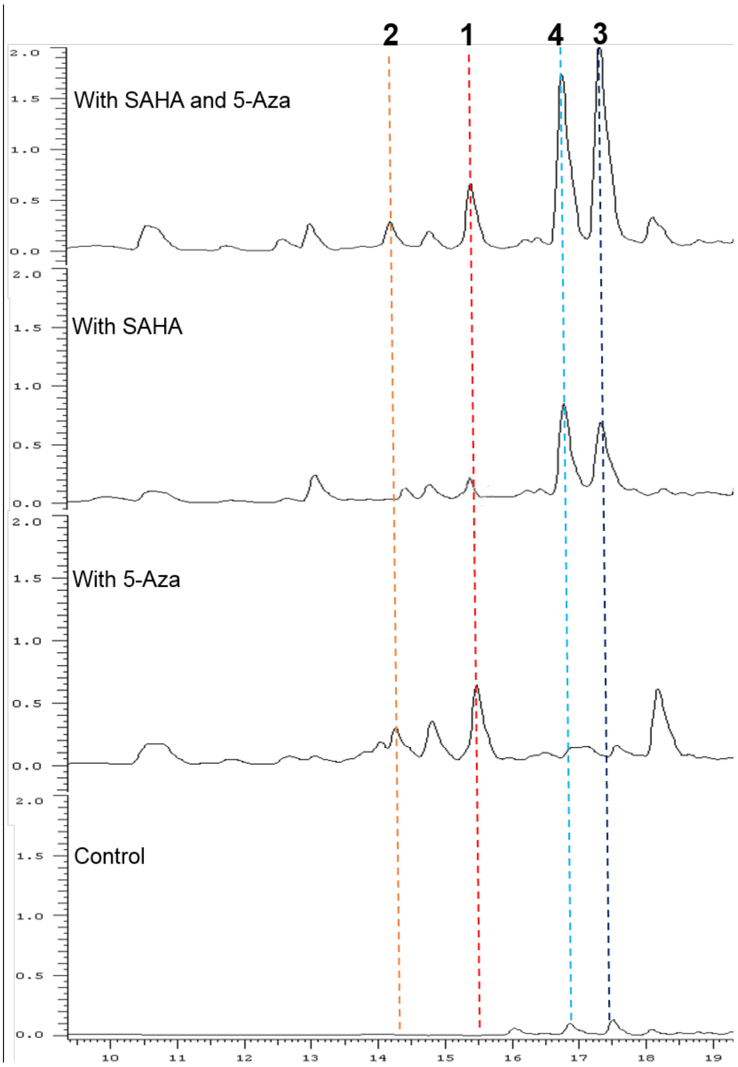

Figure S30. HPLC profiles containing compounds **1**–**4** in extracts

(HPLC chromatogram conditions: C_{18} column (Kromasil 250 mm × 4.6 mm, 5 μm) using a gradient of 15–50% acetonitrile in H_2O with 0.1% formic acid for 30 min and further up to 100% in 5 min and hold for 5 min at the flow rate of 0.8 mL/min, and recorded at 254 nm, with 20 μL of injection.)

Table S1 The contents of compounds **1**–**4** in extracts of fungal strain [a]

Culture	**1**		**2**		**3**		**4**	
	Peak area	Content [b]	Peak area	Content	Peak area	Content	Peak area	Content
With SAHA and 5-Aza	806177	1.73	1663460	10.63	9994976	186.24	16325405	424.36
With SAHA	– [c]	–	–	–	5540612	102.33	6369022	166.16
With 5-Aza	854559	2.11	1918557	12.50	–	–	–	–
Control	–	–	–	–	136151	0.51	165222	5.27

[a] The contents were calculated based on the standard curves and standard equations.

[b] The contents were recorded in mg/L.

[c] No detection due to the low contents.
